# Bioengineered immunocompetent preclinical trial-on-chip tool enables screening of CAR T cell therapy for leukaemia

**DOI:** 10.1038/s41551-025-01428-2

**Published:** 2025-07-01

**Authors:** Chao Ma, Huishu Wang, Lunan Liu, Ruiqi Chen, Nandana Mukherjee, Jie Tong, Shadab Kazmi, Xiangyi Fang, Matthew T. Witkowski, Iannis Aifantis, Saba Ghassemi, Weiqiang Chen

**Affiliations:** 1 Department of Mechanical and Aerospace Engineering, Tandon School of Engineering, New York University, Brooklyn, NY USA; 2 Department of Biomedical Engineering, Tandon School of Engineering, New York University, Brooklyn, NY USA; 3 Center for Cellular Immunotherapies, Perelman School of Medicine at the University of Pennsylvania, Philadelphia, PA USA; 4 Department of Pathology and Laboratory Medicine, Perelman School of Medicine at the University of Pennsylvania, Philadelphia, PA USA; 5 Department of Bioengineering, School of Engineering and Applied Science at the University of Pennsylvania, Philadelphia, PA USA; 6 Department of Pathology, NYU Grossman School of Medicine, New York, NY USA; 7 Perlmutter Cancer Center, NYU Grossman School of Medicine, New York, NY USA; 8 Department of Pediatrics, University of Colorado Anschutz Medical Campus, Aurora, CO USA; 9 Present Address: Center for Immunotherapy and Precision Immuno-Oncology, Lerner Research Institute, Cleveland Clinic Foundation, Cleveland, OH USA

**Keywords:** Biomedical engineering, Cancer models, Cancer immunotherapy

## Abstract

Chimeric antigen receptor (CAR) T cell immunotherapy is promising for treatment of blood cancers; however, clinical benefits remain unpredictable, necessitating development of optimal CAR T cell products. Unfortunately, current preclinical evaluation platforms are inadequate owing to their limited physiological relevance to humans. Here we engineer an organotypic immunocompetent chip that recapitulates microarchitectural and pathophysiological characteristics of human leukaemia bone marrow stromal and immune niches for CAR T cell therapy modelling. This leukaemia chip empowers real-time spatiotemporal monitoring of CAR T cell functionality, including T cell extravasation, recognition of leukaemia, immune activation, cytotoxicity and killing. We use our chip to model clinically observed heterogeneous responses such as remission, resistance and relapse under CAR T cell therapy and map factors that drive therapeutic success or failure. Finally, we demarcate functional performance of CAR T cells produced from different healthy donors and patients with cancer, with various CAR designs and protocols, systematically and multidimensionally. Together, our chip introduces an enabling ‘(pre-)clinical-trial-on-chip’ tool for CAR T cell development, which may translate to personalized therapies and improved clinical decision-making.

## Abstract (web summary)

An ex vivo organotypic and immunocompetent human leukaemia chip system is used for CAR T cell therapy modelling and screening of patient response.

## Main

Adoptive chimeric antigen receptor (CAR) T cell transfer has emerged as an encouraging immunotherapy for relapsed and refractory hematological malignancies, such as B cell acute lymphoblastic leukaemia (B-ALL)^1–3^, diffuse large B cell lymphoma^4,5^ and multiple myeloma^6–8^. However, therapeutic outcomes vary across clinical trials, for example, nearly half of patients yield to disease relapse with years of follow-up besides the side effects of cytokine release syndrome and immune effector cell-associated neurotoxicity syndrome^9–11^. Several mechanisms including T cell dysfunction^12–14^, surface antigen loss, downregulation, and mutation^15–17^, lineage switch^18,19^ and impaired death ligand^20^ that may lead to treatment failures have been reported, yet there are currently no effective molecular biomarkers that can predict patient responses. To prevent these setbacks and risks during CAR T cell development and clinical treatment, biopharmaceutical researchers need an advanced preclinical testing platform that delivers human relevant results.

Conventional strategies for preclinical assessment of CAR T cell function rely on in vitro and in vivo models^21–23^. Current in vitro assays such as two-dimensional (2D) cell co-cultures and three-dimensional (3D) tumour spheroids/organoids can assess CAR T cell function, in terms of T cell activation, cytokine secretion and tumour killing. Yet, a systematical and spatiotemporal evaluation of CAR T cell performance within the tumour niche (both tumour-associated vascular, stroma and immune components), such as CAR T cell trafficking and reciprocal microenvironmental interactions, is unattainable. These in vitro platforms may thus demonstrate limited predictive value and are mostly useful for CAR T cell development at an early stage. In vivo animal models have been widely established for preclinical cancer research; however, most of them are either immunodeficient or differing from human immunity, dictating the pursuit of humanized models^21–24^. Critically, current humanized animal models are painstaking owing to extensive preparation and subsequent month-long experiments and thwart real-time and in situ monitoring of CAR T cell response and its interaction with tumour-associated human stroma. It thus demands a reliable precision immuno-oncology tool that enables rapid, in-depth evaluation of CAR T cell therapy within a human pathophysiologically relevant context^25–27^.

In this study, we developed an ex vivo organotypic and immunocompetent human leukaemia microphysiological system exemplifying a bona fide leukaemia niche integrative of both bone marrow stroma and immune cells for CAR T cell therapy modelling and screening. Through on-chip real-time live cell imaging, comprehensive proteomic and secretomic profiling, and high-throughput single-cell mRNA sequencing (scRNA-seq), we precisely captured systematical and spatiotemporal dynamics of CAR T cell treatment, ranging from T cell extravasation, immune activation and T cell cytotoxicity to T cell killing, within human leukaemia bone marrow niche. Also, our bioengineered chip accurately validated functional performance of CAR T cells with different CAR designs and manufacturing protocols and those produced from patients with cancer, and comprehensively delineated optimal CAR T cell products using a matrix-based analytical and functional index. This preclinical platform together enables a precise, reliable and multidimensional evaluation of CAR T cell therapy, which can be readily extended to evaluate many other immunotherapies for different blood cancers as well as solid tumours and beyond.

## Results

### Engineering an ex vivo immunocompetent human leukaemia chip

**Fig. 1:**
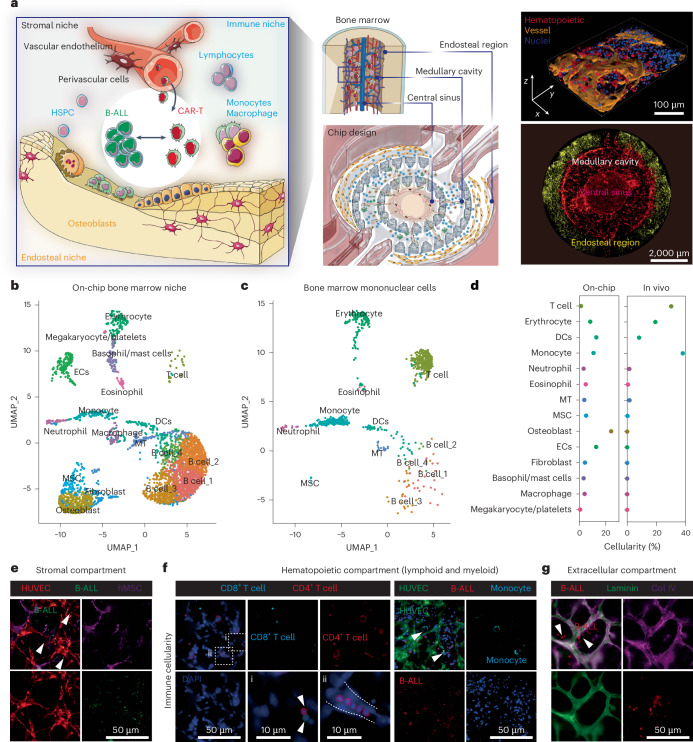
Bioengineering an ex vivo organotypic and immunocompetent leukaemia bone marrow niche chip. **a**, The compartmentalized bone marrow chip (middle) was populated with human bone marrow cells (right) to replicate the in vivo counterpart (left). The whole scanning of the leukaemia bone marrow chip (bottom right) with central sinus (RFP-HUVECs in red), medullary cavity and endosteum (DiD-labelled osteoblasts in yellow). The 3D view of perivascular niche (top right) in bone marrow with hematopoietic cells (CD45^+^ in red), vascular cells (CD31^+^ in orange) and nuclei in blue (DAPI). Representative images were from one of the three technical replicates with similar results (*n* = 3). Schematic (left part) was adapted from smart.servier.com. Schematic (right part) was created in BioRender (C.M. (2025), https://BioRender.com/wlbhoxu), **b**, scRNA-seq profiling of the bone marrow cellularity on-chip, highlighting the presence of most hematopoietic (immune) and non-hematopoietic (bone marrow stroma) cells. **c**, scRNA-seq profiling of primary bone marrow mononuclear cells, which was comparatively mapped to that of on-chip bone marrow niche. **d**, The cellularity where B cell populations were excluded to reveal bone marrow niche immune populations. In addition to the bone marrow stromal cell populations seeded to build the stromal environment, macrophage, basophil/mast cells and megakaryocyte/platelets were generated on-chip during culture. **e**, The presence of stromal compartment. HUVECs in red (RFP), Reh B-ALL cells in green (GFP) and mesenchymal cells in purple (DiD labelling). The white arrowheads indicate Reh B-ALL cells. **f**, The presence of hematopoietic cells. Lymphoid (left): CD8^+^ T cells in cyan and CD4^+^ T cells in red. The top white arrowheads indicate CD4^+^ T cells, and the bottom arrowheads indicates CD8^+^ T cells. Myeloid (right): monocyte (CD14^+^) in cyan, HUVECs (CD31^+^) in green and Reh B-ALL cells (CD19^+^) in red. The white arrowheads indicate monocytes. **g**, The deposition of ECMs such as laminin (green) and collagen IV (purple). Reh B-ALL cells in red. The white arrowheads indicate Reh B-ALL cells. Representative images were from one of the three technical replicates with similar results (*n* = 3). Source data

**Extended Data Fig. 1:**
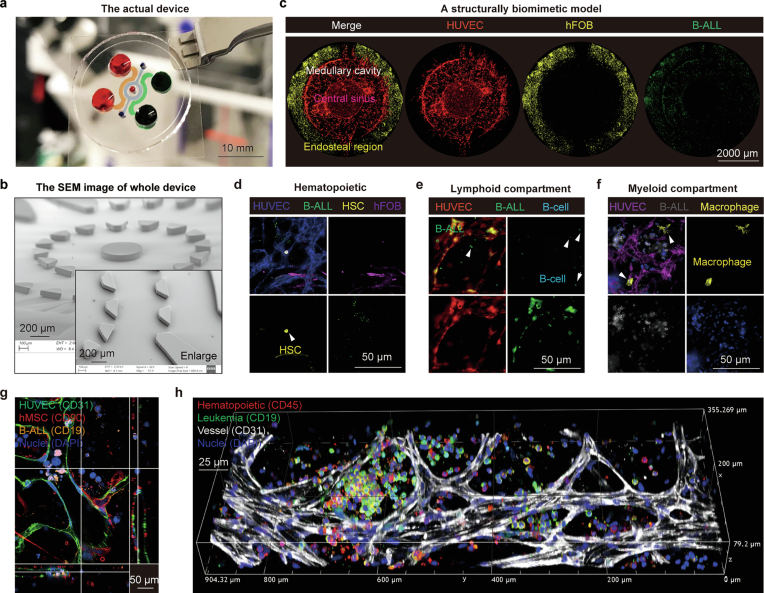
Design and fabrication of leukaemia bone marrow niche chip. (**a**) The actual chip of human leukaemia bone marrow where chambers and channels were filled with food dyes. (**b**) The scanning electron microscopy images of detailed design of the microfluidic chip. (**c**) The whole scanning of the leukaemia bone marrow niche on-chip, where RFP-expressing HUVCEs form a vascular network (red), hFOB1.19 osteoblast (yellow) and fibroblast establish an endosteal region, and Reh B-ALL cells (green) sparsely distribute throughout the niche. (**d**) Representative images showing the presence of hematopoietic stem cells (CD34^+^) in yellow in the endosteal niche. (**e**) Representative images showing the presence of B cell (CD19^+^ without GFP) and B-ALL blasts (CD19^+^ and GFP). (**f**) Representative images showing the presence of macrophages (CD68^+^) in yellow. (**g**) The 3D cross-section view of the stromal compartment. Vascular cells in green (CD31^+^), bone marrow stem/stromal cells in red (CD90^+^), leukaemia blasts in yellow (CD19^+^), and nuclei in blue (DAPI). (**h**) The 3D view of the vascular niche. Hematopoietic cells in red (CD45^+^), leukaemia blasts in green (CD19^+^), vessel in white (CD31^+^), and nuclei in blue (DAPI). Representative images were from one of three technical replicates with similar results (n = 3).

**Extended Data Fig. 2:**
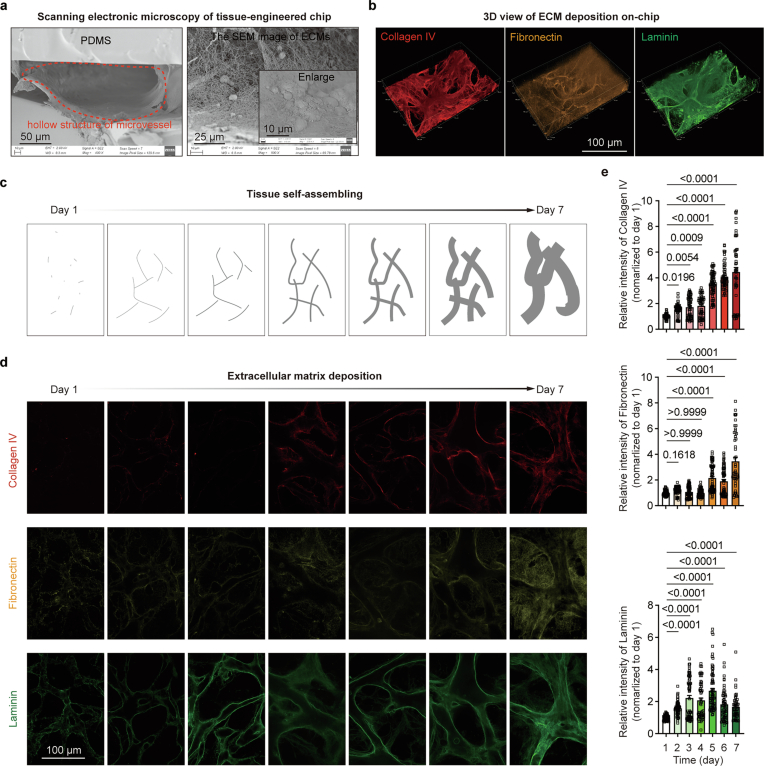
Cell self-assembling and tissue remodeling. (**a**) Scanning electronic microscopy images of tissue-engineered chip after 7-day culture. The hollow structure of microvessel formed during on-chip culture (red dash line, left) and extracellular matrix where bone marrow cells are embedded (right). (**b**) Representative images showing 3D view of key ECM components (collagen IV, fibronectin, and laminin) deposited by bone marrow niche cells on-chip after 7-day culture. (**c**) The schematic showing cell self-assembling. (**d**) Representative images showing time-lapse ECM deposition by bone marrow cells during 7-day culture. Representative images in **a**, **b**, **d** were from one of three independent experiments with similar results (n = 3). (**e**) Quantification of ECM deposition. Collagen IV (top), Fibronectin (middle), and Laminin (bottom). Images were collected from five technical replicates (n = 5) and processed with CellProfiler (version 4.1.3). One-way ANOVA followed by Kruskal-Wallis’ test, mean and s.e.m. Source data

To fill the biological and technical gaps in delivering an in-depth evaluation of CAR T cell therapy dynamics within a human relevant context, we established an ex vivo vascularized and immunocompetent human leukaemia bone marrow niche on-chip. Anatomically, leukaemia bone marrow is a multi-compartmental milieu divided into regions of central sinus, medullary cavity and endosteum, where dysfunctionally orchestrated interactions between leukaemia blasts and hematopoietic and non-hematopoietic niche cells in 3D extracellular matrices (ECMs) maintain disease progression and promote therapy resistance (Fig. 1a, left). To replicate the in vivo tissue architecture in vitro, our microfluidic chip is designed with three interconnected structural regions, including a central sinus linked to concentric medullary cavity encircled by an outer endosteal region (Fig. 1a, middle, and Extended Data Fig. 1a,b), and fabricated using replica moulding of polydimethylsiloxane (PDMS) on a glass coverslip, following our previous protocols^28–30^. The chip was populated with human hematopoietic cells (primary human bone marrow mononuclear cells), stromal cells (primary human vascular cells, mesenchymal stem cells, osteoblasts and fibroblasts) and leukaemia blasts within compartmentalized fibrin hydrogels inside which the seeded bone marrow stromal cells rebuilt and supported the three concentric tissue structures (Methods). We next exploited the de novo vascularization process of vascular cells under guidance from intrinsic and extrinsic cues such as vascular growth factors and ECMs to vascularize the tissue structure as reported previously^31–33^. In brief, the cell-laden leukaemia chip was cultured with a myriad of cytokines (maintaining hematopoietic cells and promoting vasculogenesis) for 5–7 days during which vascular cells self-assembled into a perfusable vascular network aligned by stromal cells throughout the 3D matrix (Fig. 1a, right, and Extended Data Fig. 1c–h). Hematopoietic cells and leukaemia blasts sparsely distributed within the peri-/vascular and endosteal niches, which well matched the in vivo cellular localization and composition (Extended Data Fig. 1d–h). Notably, bone marrow stromal cells (mostly vascular cells) progressively deposited supplementary ECMs such as laminin, fibronectin and collagen IV around the vasculature and throughout the niche (Extended Data Fig. 2), highlighting cell-autonomous remodelling of engineered tissue into a more biomimetic one^34–36^.

To validate the biomimicry of bioengineered bone marrow niche to its in vivo counterpart, we next utilized scRNA-seq technique to comparatively map cellularity in the sample of cells collected from our chip and matched sample of unloaded fresh bone marrow mononuclear cells (Methods, Fig. 1b,c and Supplementary Fig. 1). The scRNA-seq results validated that our bone marrow chip, even after 9-day culture, maintained an enriched cellularity with hematopoietic and stromal cells, comparable to that of the in vivo bone marrow (Fig. 1d) and keeping with recent scRNA-seq studies of the bone marrow microenvironment reported by us and others^28,37–40^. In addition, various types of bone marrow mononuclear cell demonstrated different dynamics during on-chip culture (Fig. 1b–d). For example, we observed a reduction in monocyte along with the emergence of macrophage, which may be due to that monocyte differentiates to macrophage during in vitro culture as observed previously^41^, while other myeloid cells such as neutrophil were well maintained or replenished, thanks to the on-chip reconstituted hematopoietic molecular and cellular environments. Of note, on-chip culture saw a shrinkage in T cell population owing possibly to limited replenishment of T cell as its maturation undergoes in the thymus but not bone marrow. We further corroborated this scRNA-seq analysis with immunostaining and found that most types of myeloid (for example, CD14^+^ monocytes and CD68^+^ macrophages) and lymphoid (for example, CD8^+^ and CD4^+^ T cells) cells were maintained on-chip along with the presence of the vascular network (Fig. 1e–g and Extended Data Fig. 1). Together, our tissue-engineered leukaemia chip recreates in vitro microarchitectural organization and pathophysiological signature of both human leukaemia bone marrow stroma and immune niches in vivo, enabling CAR T cell therapy to be modelled and assessed on-chip systematically and multidimensionally.

### Modelling CAR T cell therapy on-chip

**Fig. 2:**
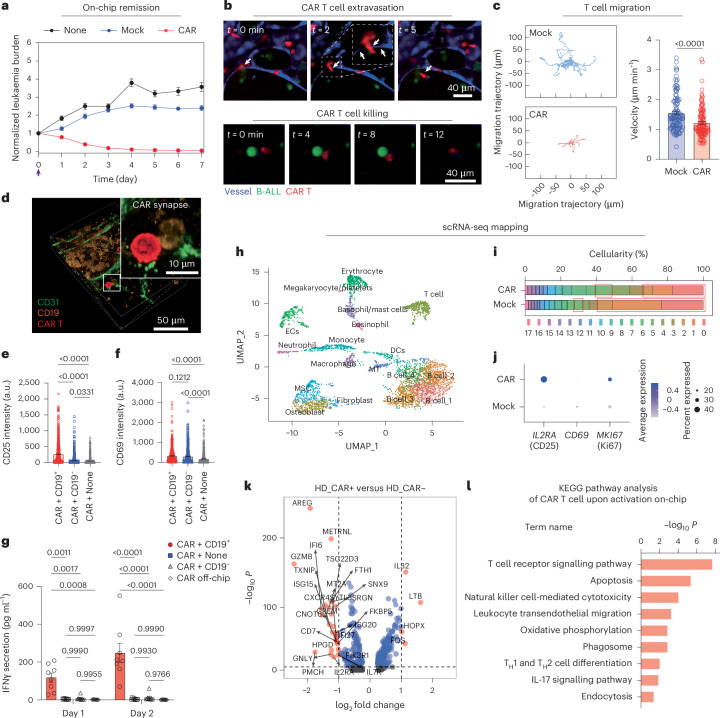
Monitoring CAR T cell dynamics in the leukaemia bone marrow niche on-chip. **a**, Leukaemia burdens of leukaemia chips treated either with 10,000 of CAR or Mock T cells (effector-to-tumour cell ratio = 1:1), or left untreated. Data were collected from four technical replicates (*n* = 4), mean and s.e.m. **b**, Representative images showing CAR T cell extravasation (top) and killing of B-ALL (bottom). The white arrows indicate T cells. Representative images were from one of the three technical replicates with similar results (*n* = 3). **c**, Comparison of T cell migration dynamics. Representative trajectories of 10 Mock T cells (top left) and 10 CAR T cells (bottom left) monitored for a 4 h period after on-chip infusion for 2 days. Data (right) were collected from four technical replicates (*n* = 4) with >100 T cells. Unpaired two-sided, Student’s *t*-test, mean and s.e.m. **d**, The formation of synapse between CAR T cell and leukaemia cell (CD19^+^ in orange) on-chip surrounded by CD31^+^ vascular cells. Representative images were from one of the three technical replicates with similar results (*n* = 3). **e**, Surface expressions of T cell activation marker CD25 on CAR T cell in different groups on day 2. Data were collected from six independent experiments (*n* = 6). One-way ANOVA followed by Tukey’s post hoc test, mean and s.e.m. **f**, Surface expressions of T cell activation marker CD69 on CAR T cell in different groups on day 2. Data were collected from six independent experiments (*n* = 6). One-way ANOVA followed by Tukey’s post hoc test, mean and s.e.m. **g**, ELISA measured secretions of IFNγ from leukaemia chips on day 1 and day 2. Data were collected from eight independent experiments (*n* = 8). Two-way ANOVA followed by Tukey’s post hoc test, mean and s.e.m. **h**, scRNA-seq mapping of bone marrow niche treated with CAR T cell on-chip for 2 days. **i**, Cellularity of samples collected from leukaemia chips treated with CAR or Mock T cell. Red boxes indicate CD19-expressing populations. **j**, Dot plot representation of mRNA expression levels of *IL2RA*, *CD69* and *MKI67* in CAR and Mock T cell. **k**, Analysis of DEGs in CAR T cells before (off-chip) and after activation (on-chip). Two-sided Wilcoxon rank sum test. **l**, KEGG pathway analysis revealed enhanced signalling pathways in CAR T cells before (off-chip) and after activation (on-chip). Two-sided Wilcoxon rank sum test. Source data

**Extended Data Fig. 3:**
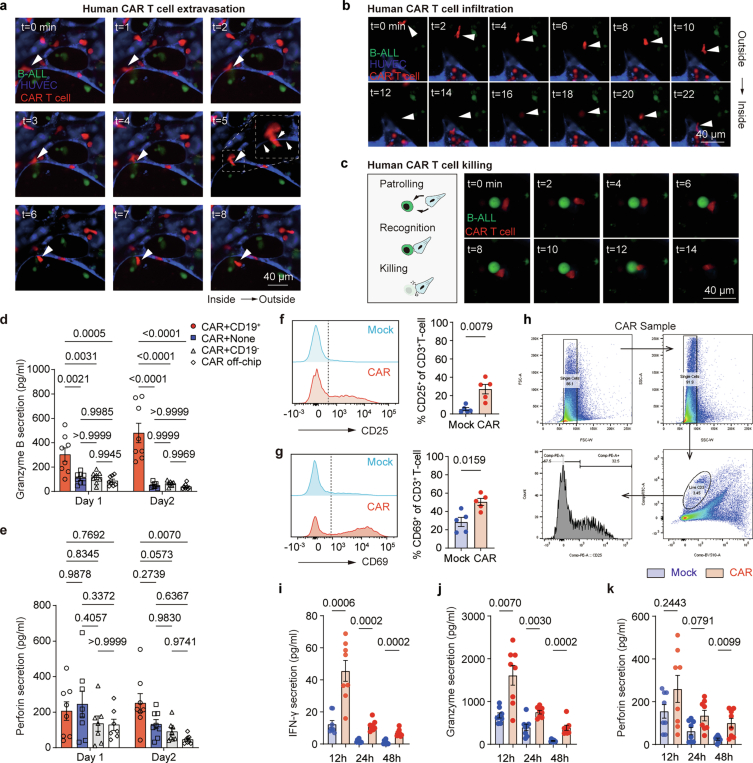
Monitoring CAR T cell dynamics on-chip. (**a-c**) Representative images showing CAR T cell extravasation (**a**), infiltration (**b**), and the process of killing a Reh B-ALL cell by a CAR T cell. CAR T cell in red, Reh B-ALL in green, and vessel (HUVECs) in blue. Representative images were from one of three independent experiments with similar results (n = 3). (**d,e**) ELISA measured CAR T cell secretions of granzyme B (**d**) and perforin (**e**) on day 2 from leukaemia chips established with CD19-expreesing leukaemia (CAR + CD19^+^), non-CD19 expressing leukaemia on-chip (CAR + CD19^-^), or no leukaemia (CAR+None) or from CAR T cell cultured off-chip (CAR off-chip). Data was collected from eight independent experiments (n = 8). Two-way ANOVA followed by Tukey’s post hoc test, mean and s.e.m. (**f**,**g**) Flow cytometry measurement of surface expressions of T cell activation markers CD25 (**f**) and CD69 (**g**) on CAR T cell and mock T cell after on-chip infusion for 2 days. Data was collected from five independent experiments (n = 5). Unpaired, two-sided, Student’s t-test, mean and s.e.m. (**h**) Gating strategy for identification of live CD3 + T cell using CAR T cell sample as an example. (**i-k**) ELISA measured secretions of INF-γ (**i**), granzyme B (**j**), and perforin (**k**) from leukaemia chips respectively treated with CAR T cell (CAR) and Mock T cell (Mock) at different time points (12 h, 24 h, and 48 h). Data was collected from four independent experiments (n = 4). Unpaired, two-sided, Mann-Whitney test, mean and s.e.m. Source data

We then modelled anti-CD19 CAR T cell therapy on-chip within a human pathophysiologically relevant context and monitored CAR T cell dynamics in a real-time manner (Methods, Fig. 2, Extended Data Fig. 3 and Supplementary Figs. 2 and 3). We first infused 10,000 of second generation (2nd-gen) human anti-CD19 4-1BBζ-CAR T cells (hereafter CAR T cell, unless stated otherwise) per chip into the perfusable vessels from central sinus and quantified the count of pre-seeded leukaemia blasts on-chip (GFP-expressing Reh, unless stated otherwise) daily for over 7 days. The results showed that CAR T cell killed ~70% of leukaemia blasts on-chip after 3 days and achieved complete eradication (>99%) of leukaemia blasts around 7 days with a 1:1 effector-to-tumour cell ratio, whereas leukaemia chips either treated with non-transduced T cell (referred as to Mock T cell) or left untreated (referred as to None) showed no control over leukaemia progression (Fig. 2a). We first investigated the impact of vascular networks on CAR T cell distribution dynamics in the leukaemia bone marrow chips with or without vasculature. While vascularized chips initially facilitated faster CAR T cell trafficking throughout the niche compared with avascularized chips, avascularized chips enhanced late-stage CAR T cell distribution (Supplementary Fig. 2), owing possibly to early interactions with leukaemia blasts boosting the expansion of CAR T cells without getting extravasated. Such a difference in CAR T cell distribution dynamics can hardly be revealed using conventional 2D assays where CAR T cells freely interact with leukaemia blasts in solution, or in vivo animal models of which devascularization is not feasible. This again validates the biomimicry of our vascularized leukaemia bone marrow models compared with other platforms. Then, we longitudinally charted the migratory behaviours of individual CAR T cells (labelled with DiD dye) in the medullary cavity region (vessel formed by RFP-expressing HUVECs) for ~12 h with fluorescence live imaging after infusion for 2–4 days (Fig. 2b and Extended Data Fig. 3a–c). We found that CAR T cells actively patrolled the leukaemia bone marrow niche such as extravasated out vessel (Fig. 2b, Extended Data Fig. 3a and Supplementary Video 1) and infiltrated into the perivascular area (Extended Data Fig. 3b and Supplementary Video 2). Notably, we captured in real time the whole process of CAR T cell killing of leukaemia at the single-cell level where a single CAR T cell moved ameboidly to reach, recognize and kill an individual leukaemia blast (Fig. 2b, Extended Data Fig. 3c and Supplementary Video 3). The migration trajectory from a 4 h observation further demonstrated different patterns between CAR T cell (mCherry expressing) and Mock T cell (DiD labelling), where CAR T cell actively patrolled and intermittently arrested at leukaemia blasts with a mean velocity of 1.213 μm min^−1^, while Mock T cell continuously patrolled at a mean velocity of 1.545 μm min^−1^ (Fig. 2c). This reduced mean velocity of CAR T cell may be due largely to its recognition of CD19^+^ leukaemia blasts and contact with the latter to form an intercellular synapse (Fig. 2d), where cytolytic granules, for example, are released. We further applied confocal microscopy to real-time monitor in 3D these cellular dynamics (for example, infiltration, extravasation and T cell killing) of CAR T cells (mCherry expressing) manufactured from a healthy donor and patient with leukaemia, respectively (Supplementary Videos 4 and 5).

We next characterized CAR T cell functionality, such as T cell activation, cytotoxicity and proliferation, after its interaction with leukaemia blasts in niche on-chip for 2 days. Upon interaction with CD19^+^ targets on-chip, CAR T cell significantly enhanced surface expression of T cell activation markers, CD25 (Fig. 2e) and CD69 (Fig. 2f), secretion of cytotoxicity-related cytokines, interferon-γ (IFNγ; Fig. 2g), granzyme B (Extended Data Fig. 3d) and perforin (Extended Data Fig. 3e), and intracellular expression of proliferation marker Ki67 (Supplementary Fig. 3), relative to on-chip CAR T cells either interacting with CD19^−^ leukaemia or without any leukaemia. These observations of T cell responses were also confirmed by comparative analysis between CAR T cell and Mock T cell (Extended Data Fig. 3f–j). Interestingly, non-CAR-expressing T cell from CAR T cell-treated chips also showed enhanced expression of T cell activation (Supplementary Fig. 3), verifying previous in vivo observation of bystander effects during CAR T cell therapy^42^. Following this, we applied an scRNA-seq technique to comparatively dissect CAR T cell and Mock T cell on-chip interacting with the leukaemia bone marrow niche for 2 days, with freshly thawed CAR T cell and Mock T cell as respective controls (Fig. 2h and Supplementary Figs. 1, 4 and 5). First, scRNA-seq results reaffirmed reduced population of CD19^+^ clusters in niche upon treatment with CAR T cell but not Mock T cell (Fig. 2i). We found that compared with freshly thawed CAR T cell, CAR T cell after on-chip activation enhanced mRNA expression of T cell activation and proliferation related genes such as *IL2RA*, *CD69* and *MKI67* (Fig. 2j and Supplementary Fig. 4a–c), validating the aforementioned observations with on-chip immunostaining. Moreover, activated CAR T cell increased mRNA expression of cytotoxicity-related genes such as *GZMB* and *GNLY* (Fig. 2k) and immune response signalling pathways such as T cell receptor signalling pathway, T_H_1 and T_H_2 cell differentiation, natural killer cell-mediated cytotoxicity and leukocyte transendothelial migration (Fig. 2l). These results together demonstrated the superiority of our leukaemia chip, compared with conventional in vitro and in vivo assays, for preclinical evaluation of functional dynamics of CAR T cell in an organotypic human leukaemia bone marrow microenvironment.

### Dissecting interaction between CAR T cell and leukaemia niche

**Fig. 3:**
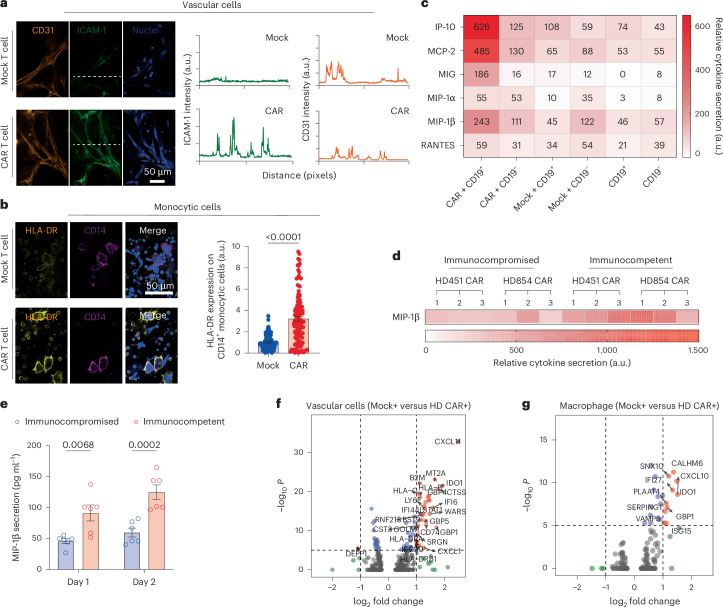
Crosstalk between CAR T cell and leukaemia bone marrow niche. **a**, Representative images showing ICAM-1 (green) expression on vascular cells (CD31^+^ in orange) from leukaemia chips treated with, respectively, Mock T cell (top left) and CAR T cell (bottom left) for 2 days. Representative images were from one of the three technical replicates with similar results (*n* = 3). Quantification data of ICAM-1 and CD31 intensity, corresponding to the white dash lines in the images. **b**, Representative images showing HLA-DR expression on monocytic cells (CD14^+^) from leukaemia chips respectively treated with Mock T cell and CAR T cell for 2 days. Representative images and data were collected from four independent experiments (*n* = 4). Unpaired, two-sided, Student’s *t*-test, mean and s.e.m. **c**, Relative cytokine secretion from leukaemia chips established with CD19^+^ or CD19^−^ leukaemia blasts, which were treated with 10,000 of CAR T cells (CAR + CD19^+^ and CAR + CD19^−^), Mock T cells (Mock + CD19^+^ and Mock + CD19^−^) or left untreated (CD19^+^ and CD19^−^). The results showed that CAR + CD19^+^ demonstrated excessive on-chip production of cytokines, such as immune stimulatory cytokines (for example, MIP-1α), chemokines (for example, MIP-1β and RANTES) and inflammatory cytokines (for example, MCP-2), among many others (Supplementary Fig. 6c). **d**, MIP-1β cytokine secretion from leukaemia chips established with (immunocompetent) or without (immunocompromised) bone marrow mononuclear cells, where MIP-1β secretion was enhanced in immunocompetent leukaemia chips. Cytokine secretion profiles in **c** and **d** were examined from chips on day 2 by using a Human Inflammation Array C3 membrane kit, and data were collected from three independent experiments (*n* = 3). **e**, ELISA quantification of MIP-1β secretion on day 2 from leukaemia immunocompetent and immunocompromised chips treated with 10,000 of CAR T cells. Data were collected from three independent experiments (*n* = 3). Two-way ANOVA, mean and s.e.m. **f**, Analysis of DEGs of vascular cells from leukaemia chips treated with CAR T cell (HD CAR^+^) or Mock T cell (Mock^+^) for 2 days. Two-sided Wilcoxon rank sum test. **g**, DEGs of macrophages from leukaemia chips treated with CAR T cell (HD CAR^+^) or Mock T cell (Mock^+^) for 2 days. Two-sided Wilcoxon rank sum test. Source data

Our understanding of the dynamic changes in the leukaemia bone marrow microenvironment during CAR T cell therapy responses such as resistance or effective killing is limited, which may prevent unleashing its full therapeutic potential^43,44^. We hypothesized that CAR T cell treatment can induce systematic immune response from the leukaemia bone marrow niche and it, in return, expands CAR T cell response. Upon on-chip infusion of CAR T cell, we found that vascular cells enhanced surface expression of intercellular adhesion molecule-1 (ICAM-1), a molecule supporting T cell extravasation (Fig. 3a), while CD14^+^ monocytic cells increased surface expression of HLA-DR, an MHC-II molecule for antigen presentation (Fig. 3b), highlighting CAR T cell-induced systematic inflammation in niche. Meanwhile, we observed an excessive on-chip production of cytokines, such as immune stimulatory cytokines (for example, MIP-1β), chemokines (for example, RANTES) and inflammatory cytokines (for example, MCP-2), among many others (Fig. 3c and Supplementary Fig. 6). This augmentation of cytokine secretion presented only in chips where CAR T cell interacted with CD19^+^ leukaemia blasts but not in those where Mock T cell interacted with CD19^+^ leukaemia blasts or CAR T cell with CD19^−^ ones (Supplementary Fig. 6c), which together confirm the specificity and efficacy of anti-CD19 CAR T cell therapy. To understand the impact of bone marrow immune cells on CAR T cell response, we further profiled the cytokine secretion signatures of leukaemia chips built with bone marrow mononuclear cells (referred as to immunocompetent) or without (immunocompromised) (Supplementary Fig. 6d). We found that secretion of MIP-1β, majorly contributed by hematopoietic cells, was higher in immunocompetent leukaemia chips than in immunocompromised ones after 2-day treatment of CAR T cell on-chip (Fig. 3d,e). Next, we applied an scRNA-seq technique to further our understanding of the response of leukaemia bone marrow niche during on-chip CAR T cell therapy at molecular and cellular levels (Fig. 3f,g and Supplementary Figs. 4 and 5). Remarkably, the scRNA-seq analysis confirmed that CAR T cell triggered systematic responses from both immune (Supplementary Fig. 4d,e) and stromal cells (Supplementary Fig. 4f,g) with altered transcriptional cascades, such as enhanced mRNA expression of interferon-induced transmembrane protein (IFITM) family members. In particular, we found that tryptophanyl-tRNA synthetase (TrpRS), *WARS*, was significantly enhanced in bone marrow stromal cells under CAR T cell therapy (Supplementary Fig. 4f,g). TrpRS not only catalyses ligation of tryptophan to its cognate tRNA during protein synthesis but also plays roles in angiogenesis, innate immunity and IFNγ signalling^45^. These results possibly indicated that the mobilized bone marrow stroma and immune niche regulated CAR T cell response, highlighting a critical role of incorporation of bone marrow niche in building preclinical systems for evaluation of CAR T cell performance. We then expanded the scRNA-seq analysis to a leukaemia patient-derived CAR T cell (PD CAR) that presented a similar profile of functional dynamics upon activation on-chip and such activated PD CAR T cell also induced a systematic response across most cell types in the leukaemia bone marrow niche (Supplementary Fig. 5). Lastly, we noticed some potential graft-versus-host effect in our allogenic system during CAR T cell therapy such as decreased CD45^+^ hematopoietic cell counts and compromised vascular network (Supplementary Fig. 6e–h). Taken together, these results demonstrated the capacity of our leukaemia chip for preclinical evaluation of functional dynamics of CAR T cell and dissection of its interaction with the leukaemia bone marrow stromal and immune niches.

### Replicating clinically relevant CAR T cell responses

**Fig. 4:**
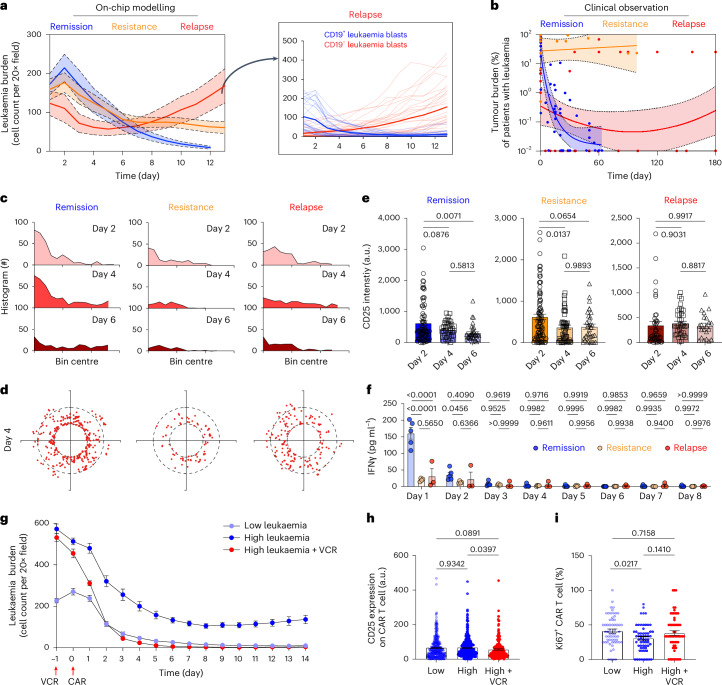
Modelling different leukaemia response scenarios post-CAR T cell therapy. **a**, On-chip monitoring of tumour burdens in leukaemia chips of different response scenarios. Data were collected from three independent experiments (*n* = 3). Inset: the counts of CD19^+^ (blue lines) and CD19^−^ (red lines) leukaemia blasts in the leukaemia bone marrow niche spiked with 5% CD19^−^ leukaemia blasts (relapse scenario). **b**, Clinical data showing tumour burdens of patients with leukaemia during CAR T cell therapy. Data were pooled and adopted from Liu et al.^50^, where different clinical studies with 209 patients were collected and unified. Nonlinear fittings were applied to remission and relapse scenarios and linear regression was applied to resistance response with 95% confidence intervals. The coloured shadings in **a** and **b** indicate the 95% confidence intervals. **c**, Histogram showing the distance of CAR T cell to chip centre on days 2, 4 and 6. Representative data were from one of the three technical replicates with similar results (*n* = 3). **d**, Dynamic distribution of CAR T cell in remission, resistance and relapse scenarios on day 4, corresponding to **c**. Each dot represents a CAR T cell. Black dash circles indicated the three concentric regions, central sinus, medullary cavity and endosteum. Representative data were from one of the three technical replicates with similar results (*n* = 3). **e**, Surface expression of CD25 on CAR T cells from different response scenarios. Data were collected from three independent experiments (*n* = 3). One-way ANOVA followed by Tukey’s post hoc test, mean and s.e.m. **f**, ELISA measured secretions of IFNγ from leukaemia chips of different response scenarios. Data were collected from three independent experiments (*n* = 3). Two-way ANOVA followed by Tukey’s multiple comparisons test, mean and s.e.m. **g**, On-chip response curves of leukaemia chips built with different initial low or high leukaemia burdens. Data were collected from eight technical replicates (*n* = 8), mean and s.e.m. **h**,**i**, Surface expression of CD25 (**h**) and nuclear expression of Ki67 (**i**) on CAR T cells in different response scenarios in **g**. Data were collected from eight technical replicates (*n* = 8). One-way ANOVA followed by Tukey’s post hoc test, mean and s.e.m. Source data

To showcase the capability of our bioengineered leukaemia chip, we modelled the processes of different clinical outcomes such as leukaemia resistance and relapse after CAR T cell therapy and mapped the underlying molecular and cellular changes (Fig. 4). Clinical diagnosis by cytomorphology sets leukaemia burden in bone marrow below 5% as remission while above 25% as relapse or resistance after treatment, although other assays such as PCR and flow cytometry take a lower percentage (0.01–0.1%) as remission^46–49^. We here followed the standard set by cytomorphology as it could allow us to quantify the number of leukaemia blasts in a single chip continuously during CAR T cell therapy with time-lapse imaging. To on-chip replicate relapse scenario from pre-existing minor CD19^−^ clones, we spiked CD19^−^ leukaemia blasts (5% or 1%) into the total leukaemia population when preparing leukaemia chips, while to mimic resistance (that is, refractory) scenario, we infused 2,500 CAR T cells per chip compared with 10,000 in the remission and relapse scenarios (Methods). Then, we monitored leukaemia burden chronologically by fluorescence imaging of on-chip CD19^+^ leukaemia blasts with GFP signal and/or CD19^−^ ones with mCherry signal every day for over 14 days. The on-chip response curves showed that 2,500 CAR T cells mostly failed to control leukaemia progression (Fig. 4a), reproducing clinical refractory cases possibly caused by insufficient expansion/persistence of patient CAR T cell products. Likewise, 10,000 CAR T cells eradicated most CD19^+^ leukaemia blasts (initial response) but spared CD19^−^ ones, which thus expanded unrestrainedly, reminiscent of clinical relapse cases driven by pre-existing CD19^−^ populations (surface antigen loss) and selective pressure from CAR T cell (Supplementary Fig. 7a,b). We next pooled and plotted clinical data of tumour burdens from 209 patients with leukaemia during CAR T cell therapy at different time points collected in our recent study^50^. As shown in Fig. 4b, some patients achieved remission with decreased leukaemia burden (remission cases), where some showed continuous leukaemia progression (resistance cases) and others achieved initial remission but unfortunately their leukaemia re-emerged several months (relapse cases) post CAR T cell therapy, confirming that our on-chip modelled different response scenarios mirrored to some extent the dynamic patterns of major clinical scenarios. Of note, although these clinical scenarios can partially be tested with conventional 2D cell culture (Methods and Supplementary Fig. 7c), CAR T cell trafficking dynamics such as T cell extravasation and migration are inherently not attainable (Supplementary Fig. 2). By contrast, our leukaemia chip can provide such information of different response scenarios, for example, CAR T cells from both remission and relapse conditions presented a pattern of wide distribution from medullar cavity to endosteum where those from resistance/refractory condition had a limited range of distribution during CAR T cell therapy on-chip (Fig. 4c,d and Supplementary Fig. 7d). Intriguingly, CAR T cell demonstrated decreased surface expression of T cell activation marker CD25 in remission and resistance groups but not in the relapse group (Fig. 4e). To identify molecular changes across different response scenarios, we further monitored dynamics of IFNγ secretion and found that IFNγ was highest in remission scenarios compared with that in resistance and relapse ones (Fig. 4f).

To further expand the clinical relevance of our system, we tested how different initial leukaemia burdens may affect CAR T cell response and whether preconditioning (for example, bridge chemotherapy) may help to improve such response. The results demonstrated that CAR T cells failed to control leukaemia progression in the chips with high leukaemia burden compared with those with low leukaemia burden given that CAR T cells were equivalently administered on-chip (Fig. 4g–i). By contrast, bridge chemotherapy (24 h treatment of 20 nM vincristine (VCR), using the protocol developed from our previous on-chip study of leukaemia stromal niche^28–30^) before administration of CAR T cells improved CAR T cell therapy response (Fig. 4g–i), replicating to some extent the effect of preconditioning during CAR T cell therapy. We also mapped the distribution patterns of CAR T cell within leukaemia chips of different tumour burdens and found that CAR T cells were more concentrated in the inner regions (near the inlet) in the leukaemia chips with high leukaemia burden than those with low leukaemia burden or preconditioned before CAR T cell loading (Supplementary Fig. 7f,g). This limited distribution of CAR T cell may in part explain the compromised leukaemia response (Fig. 4g). These results together reproduced main clinical resistance and relapse scenarios, offering us an in vitro experimental model to real-time dissect CAR T cell therapy within the whole disease spectrum.

### Validating functional performance of CAR T cell products

**Extended Data Fig. 4:**
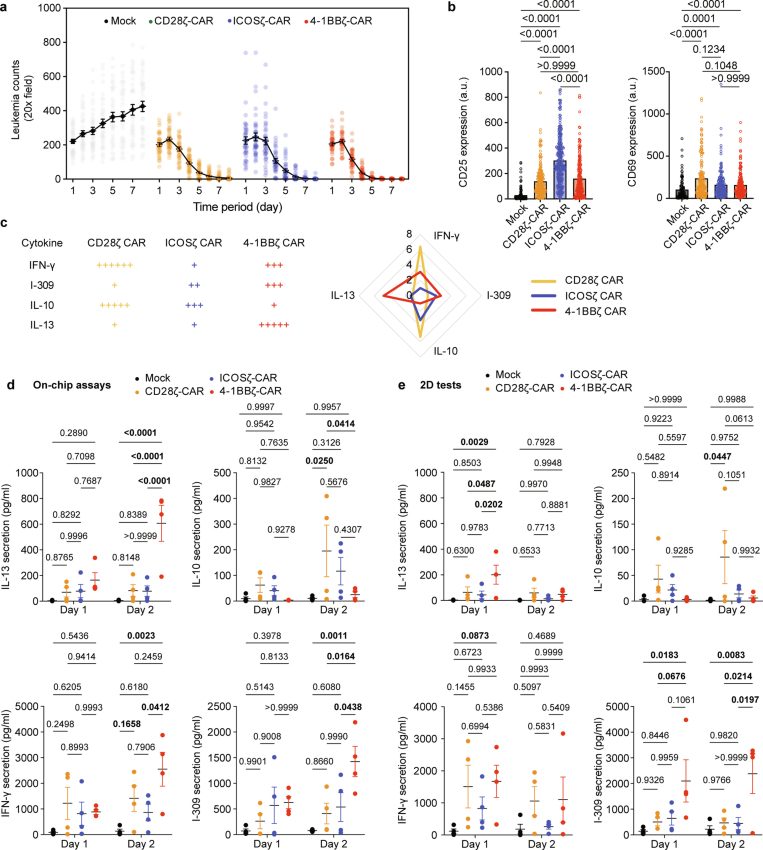
Comparative analysis of 2nd-gen CAR T cell products. (**a**) On-chip response curves of leukaemia chips under treatment of different 2nd-gen CAR T cells at a dose of 10,000 CAR T cells. Data was collected from three independent experiments (n = 3), and each had 2 or 3 chips. Data was from CAR T cell and Mock T cell of three healthy donors (ND164, ND365, and TMP497), mean and s.e.m. (**b**) CD25 (left) and CD69 (right) expression on different 2nd-gen CAR T cells activated by interaction with leukaemia on-chip for 2 days. Data was collected from two independent experiments (n = 2), and each had 2 or 3 chips. One-way ANOVA followed by Kruskal-Wallis’ test, mean and s.e.m. (**c**) Radar mapping of cytokine secretion from leukaemia chip treated with different 2nd-gen CAR T cells for 2 days. Data was normalized to that of Mock T cell, corresponding to Supplementary Fig. 8. (**d**,**e**) ELISA measurement of IL-13, IL-10, IFN-γ, and I-309 secretion from (**d**) on-chip and (**e**) 2D co-culture conditions where different 2nd-gen CAR T cells interacted with leukaemia blasts for 2 days. Data was from CAR T cell and Mock T cell of three healthy donors (ND164, ND365, and TMP497). Two-way ANOVA followed by Tukey’s post hoc test, mean and s.e.m. Source data

We next leveraged our leukaemia chip to preclinically assess the therapeutic potency of CAR T cell products generated with different designs/generations, by different manufacturing protocols, and from both healthy donors and patients with cancer. We first validated our recently developed 2nd-gen anti-CD19 CAR T cells with different designs of CAR, that is, CD28ζ-CAR, ICOSζ-CAR and 4-1BBζ-CAR, and found that these CAR T cells all achieved remission on-chip (Extended Data Fig. 4a). Also, these CAR T cells upon activation in niche on-chip enhanced surface expression of CD25 and CD69, although at different levels, compared with that of Mock T cells (Extended Data Fig. 4b). Intriguingly, cytokine profiling from on-chip tests demonstrated that 4-1BBζ-CAR secreted more interleukin (IL)-13 (a T_H_2 cytokine), and CD28ζ-CAR and ICOSζ-CAR secreted more IL-10, which may not be clearly observed in simple 2D tests with respective co-cultures of CAR T cells and leukaemia blasts (Extended Data Fig. 4c–e and Supplementary Fig. 8). Such a difference may be regulated by both intrinsic and extrinsic cues as shown in previous scRNA-seq studies that identified T_H_2 signalling deficiency as an indicator of antigen-positive leukaemia relapse post CAR T cell therapy^51,52^, clinical observations that showed that 4-1BBζ-CAR persisted longer than CD28ζ-CAR^53^, and our analysis in immunocompromised and immunocompetent leukaemia niches (Fig. 4e).

**Fig. 5:**
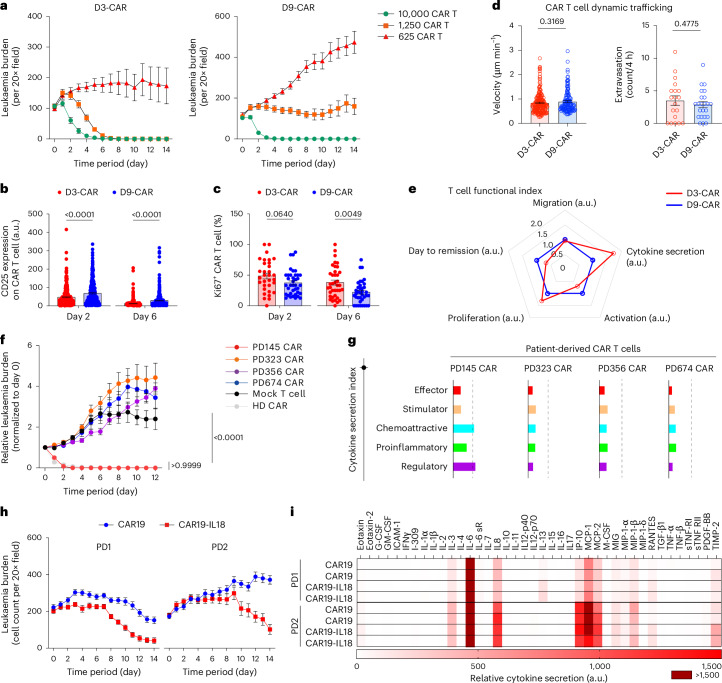
Assessing functional performance of CAR T cell products. **a**, On-chip leukaemia response curves under treatment of D3-CAR and D9-CAR T cells. Data were collected from three independent experiments (*n* = 3). **b**,**c**, Surface expression of CD25 (**b**) and nuclear expression of Ki67 (**c**) on D3-CAR and D9-CAR T cells after on-chip interaction with leukaemia blasts for 2 days with a dose of 10,000 CAR T cells. Data were collected from four technical replicates (*n* = 4). Unpaired, two-sided, Student’s *t*-test, mean and s.e.m. **d**, Comparison of T cell migration dynamics (left) and extravasation (right) of D3-CAR and D9-CAR within a 4 h monitoring period. Quantitative data were collected from three technical replicates (*n* = 3) with >100 T cells. Unpaired, two-sided, Student’s *t*-test, mean and s.e.m. **e**, Functional index of D3-CAR and D9-CAR. D3-CAR was normalized to D9-CAR, highlighting enhanced proliferation and cytokine secretion. a.u., absolute unit. Of note, the concentration of each cytokine was first normalized to that in the corresponding control group and averaged at equal weight to get the single number for cytokine secretion index. **f**, Comparative analysis of leukaemia burden on-chip treated with different patient-derived CAR T cell products (ProMab) with a dose of 10,000 CAR T cells. Data were collected from three technical replicates (*n* = 3) and normalized to day 0, mean and s.e.m. Data with statistical significance was determined by two-way ANOVA followed by Tukey’s post hoc test. **g**, Cytokine secretion index of different patient-derived CAR T cell products, benchmarked by an HD CAR T cell (dash line). PD145 CAR outperformed other PD CAR T cell products. **h**, On-chip response curves of leukaemia chips under treatment of different patient-derived CAR T cells with 2nd-gen (4-1BBζ-CAR19) or 4th-gen CAR (4-1BBζ-CAR19-IL18) designs with a dose of 625 CAR T cells. Data were collected from six technical replicates (*n* = 6). **i**, Cytokine secretion profiles of CAR19 and CAR19-IL18 on-chip (dose of 10,000 CAR T cells) on day 2 were examined by using a Human Inflammation Array C3 membrane kit with CAR T cell products of two patient donors (*n* = 2). Source data

**Extended Data Fig. 5:**
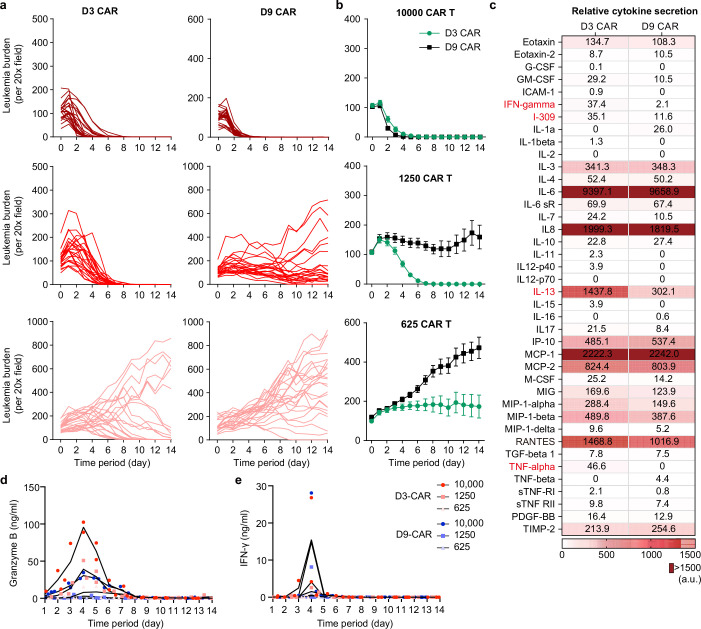
On-chip response curves under treatment of 4-1BBζ-CAR products manufactured at 3 days or 9 days (D3-CAR and D9-CAR). (**a**) D3-CAR (left) and D9-CAR (right) 4-1BBζ-CAR at different doses (that is, 10,000, 1,250, and 625 CAR T cells per chip). (**b**) Comparison between D3-CAR and D9-CAR 4-1BBζ-CAR. Data was collected from three independent experiments (n = 3), mean and s.e.m. (**c**) Cytokine secretion profiles of D3-CAR and D9-CAR 4-1BBζ-CAR on-chip (dose of 10,000 T cells) on day 2 were examined by using a Human Inflammation Array C3 membrane kit. Representative data of two independent experiments (n = 2). (**d,e**) ELISA measured secretions of granzyme B (**d**) and IFN-γ (**e**) from leukaemia chips of D3-CAR and D9-CAR treatment at different time points. Data was collected from two independent experiments (n = 2). Source data

Current CAR T cell manufacturing protocols activate and expand T cell for at least 6 days, and we previously found that activated T cells undergoing rapid proliferation drove differentiation towards effector cells with a loss of anti-leukaemic potency^54,55^. To validate whether we can circumvent such loss by shortening ex vivo culture period of CAR T cells, we on-chip compared CAR T cell products manufactured by 3 (D3-CAR) and 9 days (D9-CAR) adapting our recent CAR stress test^55^. Although both CAR T cell products at higher doses eradicated leukaemia completely (Fig. 5a and Extended Data Fig. 5a,b), D3-CAR did outcompete D9-CAR at lower doses. To understand the mechanisms underlying improved outcome, we evaluated first the expression of T cell activation markers CD25 and CD69, which unexpectedly was stronger in D9-CAR instead of D3-CAR (Fig. 5b). This difference can be partially explained by the high proliferating status of D3-CAR that showed enhanced expression of Ki67 than did D9-CAR (Fig. 5c), although T cell trafficking dynamics said comparable ability (Fig. 5d). As such, cytokine profiling revealed greater secretion from D3-CAR instead of D9-CAR (Extended Data Fig. 5c,d). Furthermore, a functional index was developed to systematically delineate and compare the potency of these two CAR T cell products, highlighting the contribution by enhanced proliferation and total cytokine secretion in D3-CAR (Fig. 5e).

**Extended Data Fig. 6:**
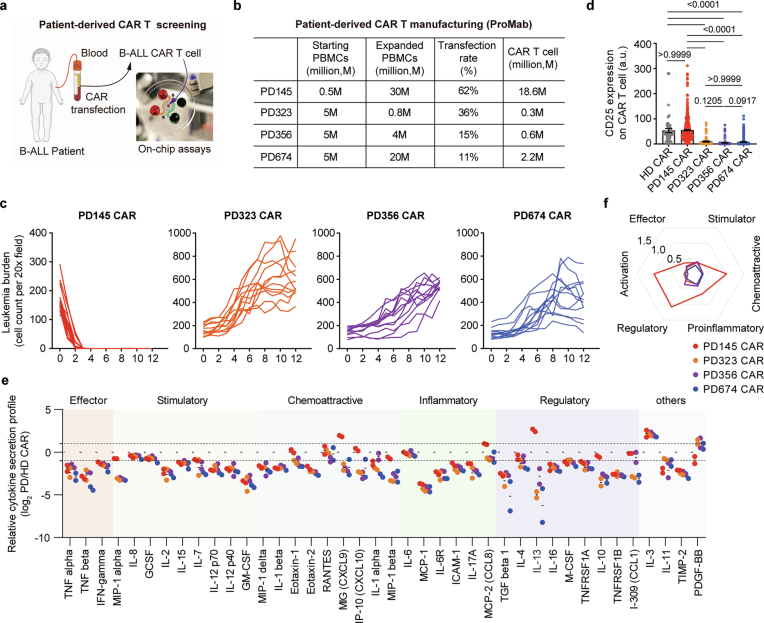
On-chip response curves under treatment of different patient derived 2nd-gen CAR T cells. (**a**) Schematic of on-chip evaluation of patient derived CAR T cell products. Created in BioRender. MA, C. (2025) https://BioRender.com/04m813s. (**b**) Manufacturing of patient derived CAR T cells, where three patients with B-ALL leukaemia (PD323, PD356, and PD674) and one control patient with lung cancer (PD145). (**c**) On-chip response curves under treatment of different patient derived CAR T cells, PD145 CAR, PD323 CAR, PD356 CAR, and PD674 CAR (ProMab) at a dose of 10,000 T cells. Data was collected from three technical replicates (n = 3). (**d**) Surface expression of CD25 on patient derived CAR T cells, PD145 CAR, PD323 CAR, PD356 CAR, and PD674 CAR, after on-chip interaction for 2 days. Data was collected from at least three technical replicates (n = 3). One-way ANOVA followed by Kruskal-Wallis’ test, mean and s.e.m. (**e**) Cytokine secretion profiles of patient-derived CAR T cell products, PD145 CAR, PD323 CAR, PD356 CAR, and PD674 CAR, on-chip at day 2 were examined by using a Human Inflammation Array C3 membrane kit. Data was collected from two independent experiments (n = 2). (**f**) Radar map depicting functional performance, that is, activation, effector, stimulator, chemoattractive, proinflammatory, and regulatory, of different PD CAR T cells. PD145 CAR outperformed other PD CAR T cell products. Source data

**Extended Data Fig. 7:**
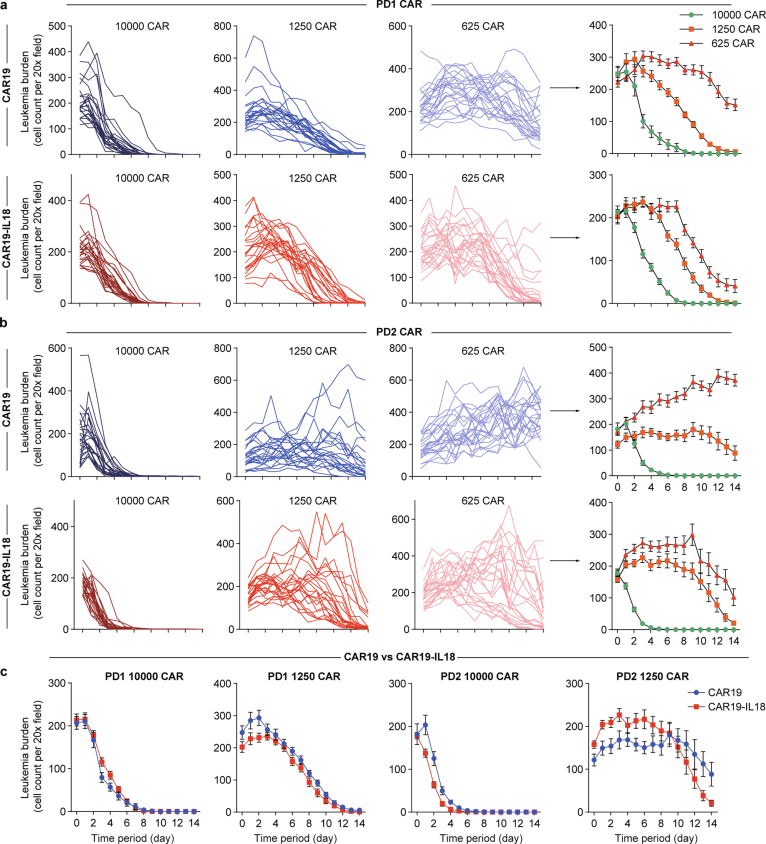
On-chip response curves under treatment of 2nd-and 4th-gen CAR T cells of two leukaemia patients. (**a,b**) Comparative analysis of 2nd-gen 4-1BBζ-CAR T cells (CAR19) and 4th-gen 4-1BBζ-CAR-IL18 T cells (CAR19-IL18) at doses of 10,000, 1,250 and 625 CAR T cells for patient donor 1 (PD1) and patient donor 2 (PD2). Data was collected from three technical replicates (n = 3). (**c**) Summary of comparative analysis of CAR19 and CAR19-IL18 T cells at 10,000 and 1,250 CAR T cells. Data was collected from six technical replicates (n = 6), mean and s.e.m. Source data

In addition, we evaluated 2nd-gen anti-CD19 4-1BBζ-CAR T cell products generated from four patients either with B-ALL leukaemia (PD323, PD356 and PD674) or non-B-ALL lung cancer (PD145) (Fig. 5f and Extended Data Fig. 6a,b). Of these four patients, on-chip remission was only achieved with treatment of PD145 CAR T cells that significantly enhanced surface expression of CD25 on day 2 (Extended Data Fig. 6c,d). To better outline the cytokine secretion, we classified cytokines into five functional categories, that is, effector, stimulatory, chemoattractive, inflammatory and regulatory, to quantify and normalize the weighted average (Extended Data Fig. 6e). Also, PD323, PD356 and PD674 CAR T cells demonstrated a notably weakened cytokine secretion index compared with that of PD145 CAR T cell, benchmarked by an HD CAR T cell (Fig. 5g). The varied outcomes can be partially explained by the limited expansion capacity of T cells from PD323, PD356 and PD674 during CAR T cell manufacturing (ProMab) as on-chip treatments were all given at a dose of 10,000 CAR T cells (Extended Data Fig. 6b). Such a difference can also be reflected by the functional index incorporating expression of T cell activation marker (Extended Data Fig. 6d), where PD145 CAR T cells outperformed other PD CAR T cells (Extended Data Fig. 6f). We then hypothesized that adding new modalities into CAR design may improve the performance of patient CAR T cells, such as incorporation of IL-18 (4th-gen CAR design), to potentiate CAR T cell function or mobilize bone marrow immunity^56^. Initial on-chip analyses using healthy donor-derived CAR T cells confirmed that 4-1BBζ-CAR-IL18 achieved a rapid remission response at high dose and surpassed 4-1BBζ-CAR in the CAR stress test (Supplementary Fig. 9a–f). Again, 4-1BBζ-CAR-IL18 showed a higher cytokine secretion than 4-1BBζ-CAR with a boosted index of cytokine secretion in all categories, including those T_H_2 cytokines (Supplementary Fig. 9g,h), as well as stronger CD25 expression upon activation on-chip (Supplementary Fig. 9i). We further tested two leukaemia patient-derived CAR T cell products: 4-1BBζ-CAR (CAR19) and 4-1BBζ-CAR-IL18 (CAR19-IL18) of PD1 and PD2, respectively. The results showed that both PD1 CAR T cell products controlled leukaemia progression, whereas PD1 CAR19-IL18 demonstrated a slight enhancement; by contrast, PD2 CAR19 was not able to achieve remission during the CAR stress test, while PD2 CAR19-IL18 prevented leukaemia expansion (Fig. 5h,i and Extended Data Fig. 7), demonstrating that 4th-gen CAR design improved on-chip response, especially for those whose 2nd-gen CAR T cell product generated suboptimal anti-leukaemia responses.

**Fig. 6:**
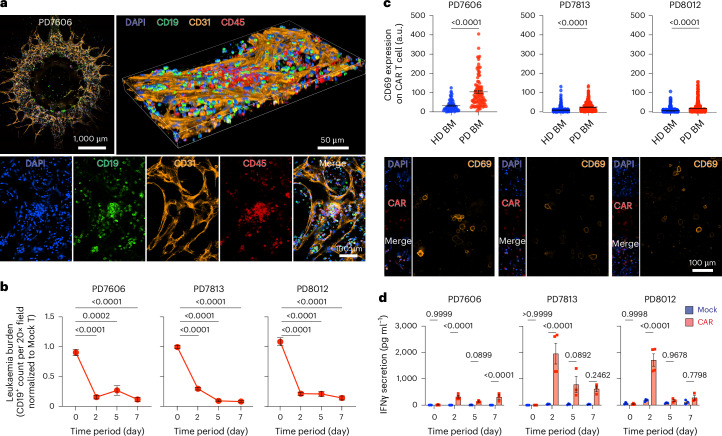
Evaluating patient-specific CAR T cell responses. **a**, The whole scanning (top left), 3D view (top right) and 2D view (bottom) of patient-specific bone marrow niche on-chip built with PD7606 bone marrow mononuclear cells after 9-day on-chip culture. Representative images were from one of the three technical replicates with similar results (*n* = 3). **b**, On-chip response curves of patient-specific leukaemia chips under treatment of donor matched 4-1BBζ-CAR T cells (dose of 10,000 CAR T cells). Three patients were tested, that is, PD7606, PD7813 and PD8012. Data were collected from three technical replicates (*n* = 3), where each chip has eight random fields quantified (*n* = 24 images). One-way ANOVA followed by Tukey’s post hoc test, mean and s.e.m. **c**, Quantification (top) and representative images (bottom) of surface expression of CD69 on CAR T cell from matched patient-specific leukaemia chips. CAR T cell from chips prepared with healthy donor-derived bone marrow mononuclear cells (HD BM) was used as the control (Extended Data Fig. 8d). Data were collected from three technical replicates (*n* = 3), where each chip has ten random fields quantified (*n* = 10 images) with >200 T cells. Unpaired, two-sided, Student’s *t*-test, mean and s.e.m. **d**, ELISA measured IFNγ secretions from patient-specific leukaemia chips treated with donor-matched CAR T cell (CAR) and Mock T cell (Mock) at different time points (days 0, 2, 5 and 7). Data were collected from three technical replicates (*n* = 3). Two-way ANOVA followed by Tukey’s post hoc test, mean and s.e.m. Source data

**Extended Data Fig. 8:**
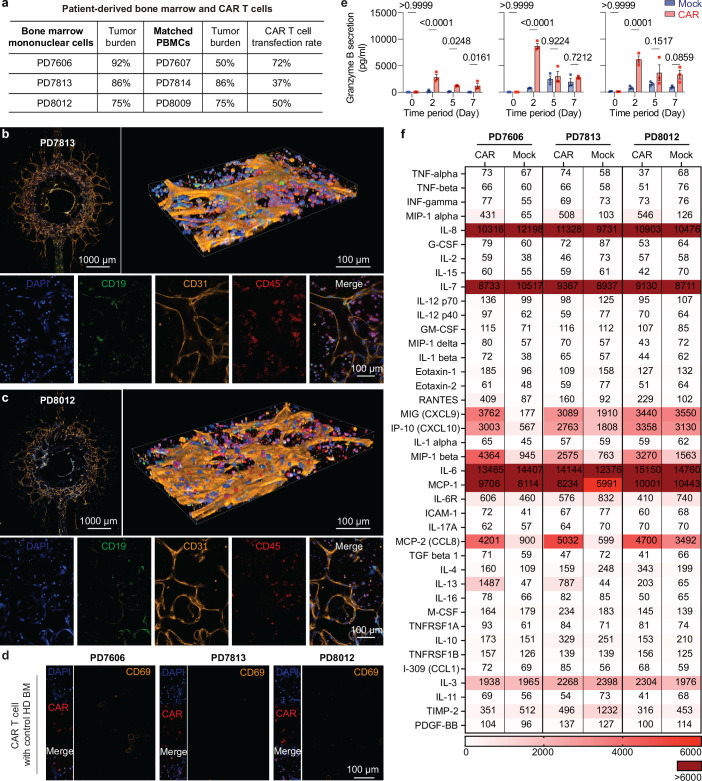
Patient-specific CAR T cell responses in donor matched leukaemia chips. (**a**) Donor matched leukaemia chip study using three matched patient samples (PD7606, PD7813, and PD8012 bone marrow mononuclear cells and matched PD7607, PD7814, and PD8009 peripheral blood mononuclear cells, respectively). (**b,c**) The whole scanning (top left), 3D view (top right), and 2D view (bottom) of patient-specific bone marrow niches on-chip built with bone marrow mononuclear cells from donors PD7813 (**b**) and PD8012 (**c**) after 5-day on-chip culture. Representative images were from one of three technical replicates with similar results (n = 3). (**d**) Representative images of surface expression of CD69 on CAR T cells from chips prepared with healthy donor-derived bone marrow mononuclear cells, corresponding to Fig. 6c. Representative data from three technical replicates with similar results (n = 3). (**e**) ELISA measured secretions of granzyme B from patient-specific leukemia chips treated with donor-matched CAR T cell (CAR) and Mock T cell (Mock) at different time points (day 0, 2, 5, and 7). Data was collected from 3 technical replicates (n = 3). Two-way ANOVA followed by Tukey’s post hoc test, mean and s.e.m. (**f**) Cytokine secretion profiles of different matched patient-derived 4-1BBζ-CAR T cells on-chip (dose of 10,000 CAR T cells) on day 2 were examined by using a Human Inflammation Array C3 membrane kit. Data was mean of two technical replicates (n = 2). Source data

Lastly, we applied our leukaemia system to evaluate patient-specific CAR T cell responses using three matched patient samples (PD7606, PD7813 and PD8012 bone marrow mononuclear cells and matched PD7607, PD7814 and PD8009 peripheral blood mononuclear cells, respectively; Methods, Fig. 6a and Extended Data Fig. 8a–c). We found that within the three patient-specific chips, 4-1BBζ-CAR T cells significantly eradicated leukaemia blasts than did Mock T cells (Fig. 6b). We confirmed that the significant decrease of leukaemia counts was mainly driven by CAR T cell activation where patient CAR T cells enhanced their surface expression of CD69 in matched leukaemia chips but not in control chips (Fig. 6c and Extended Data Fig. 8d). In parallel, IFNγ and other cytotoxic cytokines were significantly higher in CAR T cell-treated groups than Mock T cell-treated groups, whereas the levels of cytokine secretion varied strongly across patients’ CAR T cells (Fig. 6d and Extended Data Fig. 8e,f). These results highlighted the applicability of our patient-specific system for autologous CAR T cell studies. Together, our bioengineered leukaemia chip demonstrated a useful and powerful preclinical platform for CAR T cell development.

## Discussion

The past decade has witnessed the potential of microfluidic organs-on-chips as a tool for advancing immunology and immuno-oncology research. For instance, an in vitro human lymphoid follicle-on-chip with ectopic lymphoid follicle formation was recently developed and demonstrated its application in the evaluation of vaccines and adjuvants^57^. In addition, several studies have developed cancer-on-chip platforms to assess cell-based immunotherapies, for instance, T cell receptor-engineered T cell^58,59^, CAR T cell^60–62^ and NK cell^63–65^, as well as their interaction with extrinsic cues such as hypoxia, physical barriers and extracellular matrix, and other immune cells^58–69^. Thus far, the current cancer-on-chip model is in its infancy and a precision immuno-oncology screening platform that can model CAR T cell therapy with unique and complex human immune signatures is highly desirable while largely unavailable. Previously, we reported a leukaemia-on-a-chip to study chemoresistance pathways contributed by leukaemia bone marrow stroma, and using scRNA-seq we also interrogated healthy and leukaemia bone marrow microenvironments and revealed heterogeneous immune dynamics at the single-cell level across clinical response scenarios after chemotherapy^28–30,37^. Integrating these technical innovations and biological insights, we herein develop a 3D vascularized and immunocompetent leukaemia microphysiological system that recapitulates human leukaemia bone marrow stroma and immune niches.

Replicating the in vivo bone marrow microarchitecture and immunity, our leukaemia chip allows us to map the systematical and spatiotemporal dynamics of CAR T cell treatment, which is at large not offered by conventional in vitro assays. Also, we model major therapeutic response scenarios such as remission, resistance and relapse post-CAR T cell therapy observed in clinical settings and map corresponding CAR T cell dynamics, transcending current in vitro analyses that are limited to short experimental periods and static time points. The on-chip modelled resistance response reproduces clinical refractory cases driven by both inadequate CAR T cell expansion and exhaustion in CAR T cell products, while the relapse response modelled on-chip tests clonal selection for pre-existing minor CD19^−^ clones that drive relapse upon the selective pressure imposed by CAR T cell therapy. Other types of resistance or relapse scenarios presented in clinical trials such as acquired resistance through surface antigen mutation following CAR T cell challenge have not been attempted with our leukaemia chip and are directions for future study. In addition, the correlation between preclinical in vitro observations and clinical trials remains to be determined, such as the inherently present mismatched timeline of clinical and preclinical studies. The related data of clinical observations of patient dynamics are extremely limited and mostly inaccessible, preventing further studies at this time. Nevertheless, our platform can be assembled rapidly (half day) and allows at least a 14-day long evaluation of CAR T cell responses, which is more convenient compared with immunocompetent animal models that can take up to half year or longer to establish, let alone month-long experiments subsequently^21–24^.

On the basis of multidimensional and spatiotemporal measurements, we comprehensively delineate therapeutic functionality of 2nd-gen CAR T cell of different designs and confirm that a limited T_H_2 function may compromise CAR T cell performance and such a difference may be regulated by intrinsic and extrinsic cues in systematic immune response^51–53^. We also studied CAR T cells manufactured with different protocols, shortening ex vivo culture and expansion period from 9 to 3 days (D3-CAR and D9-CAR). By applying a CAR stress test, our leukaemia chip confirmed the superior efficacy and potency of D3-CAR over D9-CAR in a human relevant context. On the basis of systematical on-chip functional performance analyses, we further developed an analytical index to delineate the therapeutic potency of CAR T cells. Furthermore, we evaluated patient-derived 2nd-gen anti-CD19 CAR T cell products generated from four patients either with B-ALL leukaemia or non-B-ALL cancer using our bioengineered chip. The varied outcomes from these experiments can be partially explained by the hypofunctions (limited expansion and cytotoxicity) of patient CAR T cell products. In parallel, we demonstrated that 4th-gen CAR design by integration of new modalities such as IL-18 can strengthen the functional performance of healthy donor and leukaemia patient-derived CAR T cells, which holds promise for patients with leukaemia, especially those whose 2nd-gen CAR T cell products fail to generate robust anti-leukaemia responses. Lastly, we prepared our leukaemia system with patient-derived bone marrow mononuclear cells and evaluated on-chip patient-specific responses to CAR T cell products manufactured from matched blood samples, highlighting the translational potential of our leukaemia system. We still used healthy donor-derived bone marrow stromal cells owing to the unavailability of matched patient stromal samples. As such, graft-versus-host effect cannot be fully ruled out, as demonstrated in a recently reported blood cancer model^70^. Nevertheless, our platform as well as others can be applied to evaluate allogeneic CAR T cells that are widely pursued to offer products for clinical treatments.

Current attempts to overcome the drawbacks of CAR T cell therapy are primarily focused on persistence and specificity of effector cells. While this is undoubtedly important to maximize the potential of CAR T cell, a detailed understanding of the impact of tumour immunity on CAR T cell therapy is also indispensable^71,72^. To reconstitute the in vivo leukaemia immune milieu in vitro, we populated our leukaemia chip with primary human bone marrow mononuclear cells and confirmed that the derived systematic immune response in leukaemia niche may help to expand CAR T cell response. It is acknowledged that bone marrow mononuclear cells contain less to no granulocytes and are mostly short-lived; thus, the long-term regulatory role of these cell types in CAR T cell therapy remains to be investigated in future. In addition, several studies have recently observed the correlation between clonal hematopoiesis of indeterminate potential (CHIP) and CAR T cell immunotherapy responses, as well as the accompanying adverse reactions, such as cytokine release syndrome and immune effector cell-associated neurotoxicity syndrome^73–75^. Yet whether a causative relationship can be established remains to be investigated, and modelling and dissecting of CHIP in the leukaemia bone marrow niche can be future research directions, especially for adult patients with blood cancer, given that CHIP mutations rarely present in paediatric and adolescent patients^76–78^. Most critically, incorporation of clinical bone marrow samples (primary leukaemia blasts and both stroma and immune parts) to build a fully patient-derived screening platform is necessary to enable matched studies between preclinical functional tests and clinical performance of CAR T cell products, although the limited availability of patient samples is a critical issue, as well as the compromised viability of leukaemia blasts both after isolation and during in vitro culture. As such, patient’s induced pluripotent stem cell (iPSC)-derived various cell types can be used as surrogate as demonstrated in two recent studies where iPSC-derived bone marrow organoids were generated for disease modelling^79,80^. Additional work to combine biochemical gradients, hypoxia, ECM stiffness and interstitial flow into our leukaemia chip will further expand our understanding of the effect of other microenvironmental cues on CAR T cell therapy. Lastly, in-depth and seamless collaboration between bioengineers, oncologists and physicians will be required to enable the realization of translating such bioengineered platform from bench to bedside and beyond.

In conclusion, our tissue-engineered immune-oncology model offers an ideal precision medicine platform for preclinical evaluation of CAR T cell immunotherapies, which can accelerate preclinical CAR T cell development, bridge up biological and technical gaps between preclinical studies and clinical trials, and ultimately pave ways to reliably screen responders and non-responders and develop optimal CAR T cell therapy.

## Methods

### Cell culture and reagents

Human B-ALL cells (Reh, catalogue number CRL-8286, ATCC), CD19-expressing K562-meso-19, K562-meso-19-GFP and K562-meso-19-mCherry leukaemia cell lines provided by S. Ghassemi’s lab were cultured in RPMI medium (catalogue number 11875135, Thermo Fisher Scientific) supplemented with 10% fetal bovine serum (FBS; catalogue number A5256701, Thermo Fisher Scientific), 1% penicillin/streptomycin (catalogue number 15140163, Thermo Fisher Scientific), 100 μM l-glutamine (GlutaMAX supplement, catalogue number 35050061, Thermo Fisher Scientific) and 50 μM β-mercaptoethanol (catalogue number 21985023, Thermo Fisher Scientific) in a 37 °C incubator with 5% CO_2_. The Reh B-ALL cell line was validated by short tandem repeat analysis through ATCC. GFP stably expressing Reh cell line (catalogue number T3959) was bought from Applied Biological Materials and cultured in Prigow IV media (catalogue number TM004, Applied Biological Materials) supplemented with 10% FBS and 0.2 μg ml^−1^ puromycin (catalogue number G264, Applied Biological Materials). K562-meso-19, K562-meso-19-GFP and K562-meso-19-mCherry leukaemia cell lines were particularly used for comparatively validating the functional performances of healthy donor-derived 2nd-gen 4-1BBζ-CAR19 and 4th-gen 4-1BBζ-CAR19-IL18 T cells. Primary human umbilical vein endothelial cells (HUVECs; catalogue number C2519A, Lonza), VE-CAD-GFP-expressing HUVECs (catalogue number cAP-0001VECAD-GFP, Angio-Proteomie) and RFP-expressing HUVECs (catalogue number cAP-0001RFP, Angio-Proteomie) were cultured in Endothelial Cell Growth Medium-2 BulletKit (EGM-2; catalogue number CC-3162, Lonza) and used within passage 5. Primary human mesenchymal stem cells (hMSCs; catalogue number PT-2501, Lonza) were cultured in Mesenchymal Stem Cell Growth Medium BulletKit (MSCGM; catalogue number PT-3001, Lonza) and used within passage 5. Primary normal human lung fibroblast (NHLF; catalogue number CC-2512, Lonza) was cultured in Fibroblast Growth Medium-2 BulletKit (FGM-2; catalogue number CC-3132, Lonza) and used within passage 8. Osteoblasts were differentiated from hMSCs using Human Mesenchymal Stem Cell Osteogenic Differentiation Medium BulletKit (catalogue number PT-3002, Lonza) for at least 12 days and designated as hMSC-derived osteoblasts. Human fetal osteoblastic cell line (hFOB1.19, catalogue number CRL-11372, ATCC) was bought from ATCC and validated by short tandem repeat analysis. hFOB1.19 cells were cultured with DMEM/F12 (catalogue number 11320033, Thermo Fisher Scientific) supplemented with 10% FBS, 1% penicillin/streptomycin, 100 μM l-glutamine and 0.3 mg ml^−1^ G418 (catalogue number 10131035, Thermo Fisher Scientific). Unless stated otherwise, hFOB1.19 cells were only used for the characterization of the whole scanning of leukaemia chip. To create a physiologically relevant bone marrow immune model, commercial bone marrow mononuclear cells (catalogue number 70001.1, STEMCELL Technologies) containing various bone marrow immune cells from healthy donors were used.

Human anti-CD19 scFv-4-1BB-CD3ζ CAR (4-1BBζ-CAR, for short) T cells from healthy donors (HD) and patients with cancer (PD; PD145, PD323, PD356 and PD674) or non-engineered (Mock) T cells were purchased from ProMab Biotechnologies by a customized order with expansion for about 10 days. Unless otherwise stated, 2nd-gen human anti-CD19 CAR T cells (that is, CD28ζ-CAR, ICOSζ-CAR, 4-1BBζ-CAR, and D3 and D9 4-1BBζ-CAR (D3-CAR and D9-CAR)), 4th-gen 4-1BBζ-CAR-IL18 T cells and 4-1BBζ-CAR T cells of patients with leukaemia (PD7607, PD7814 and PD8009), as well as respective Mock T cells, were prepared by S. Ghassemi’s lab at University of Pennsylvania School of Medicine with expansion for about 10 days. The anti-CD19 4-1BBζ-CAR T cells and autologous 4-1BBζ-CAR-IL18 T cells of patients with leukaemia were obtained from a huCART19-IL18 clinical trial at the University of Pennsylvania (ClinicalTrials.gov NCT04684563). 4-1BBζ-CAR-IL18 T cells were produced with a humanized anti-CD19 CAR lentiviral vector that was engineered to co-express IL-18. The study was approved by the Institutional Review Board at the University of Pennsylvania. It was conducted in accordance with the principles of the Declaration of Helsinki. All patients provided written informed consent. To ensure compliance with the HIPAA regulations, all of the samples were deidentified before analysis on-chip. Before on-chip loading, CAR T cells and Mock T cells were thawed and recovered overnight in ImmunoCult-XF T Cell Expansion Medium (catalogue number 10981, STEMCELL Technologies) supplemented with 200 U ml^−1^ recombinant human IL-2 (catalogue number 200-02, Peprotech).

### Preparation of leukaemia chip

The 3D microfluidics-based organotypic leukaemia bone marrow niche chip is composed of three distinct functional regions (Fig. 1 and Extended Data Fig. 1), including a central sinus region vascularized by endothelial cells, an inner ring region that functions as bone marrow cavity populated with bone marrow mononuclear cells, stromal cells and leukaemia, and the outer ring channels, which serve as the endosteal region inoculated with osteoblasts and fibroblasts and connects with four media reservoirs, which are for cell culture media supply and waste removal. The microfluidic chip was fabricated using a standard soft lithography technique by replica moulding of PDMS (Sylgard184, catalogue number DC2065622, Dow Corning) from a master mould and bounded onto a glass coverslip (22 × 22 mm, catalogue number CLS285022, Thermo Fisher Scientific)^28–30^. The master mould for the microfluidic chip was fabricated with SU-8 negative photoresist (catalogue number SU8-2050, Microchem) at a thickness of 100 μm on a silicon wafer (catalogue number 452, University Wafer) by using photolithography. Once PDMS replica was made from the master mould, 1 mm, 1.5 mm and 4 mm holes were punched for two side inlets, one central inlet and four outlets, respectively, before it is bounded to the coverslip. Before cell loading, microfluidic chips were treated under ultraviolet for sterilization in a type 2 class laminar flow hood for 20 min.

To prepare the leukaemia chip model, human leukaemia blasts, bone marrow stromal cells and bone marrow immune cells were loaded into the microfluidic chips with physiologically relevant seeding density for each cell type (that is, HUVECs at 1 × 10^7^ cells per ml, hMSCs at 1 × 10^5^ cells per ml, NHLF at 2 × 10^6^ cells per ml, hMSC-derived osteoblasts at 2 × 10^6^ cells per ml, Reh B-ALL at 1 × 10^6^ cells per ml and bone marrow mononuclear cells at 5 × 10^6^ cells per ml) in a fibrin hydrogel. In general, a multiple-step loading protocol was followed to compartmentalize HUVECs in the centre of the chip, HUVECs, hMSCs, Reh B-ALL and bone marrow mononuclear cells in the perivascular area, and NHLF and hMSC-derived osteoblasts in the endosteal region. Unless stated otherwise, CD19-expressing K562 leukaemia cell lines were used with a seeding density at 5 × 10^5^ cells per ml for 4th-gen CAR T cell testing experiments. First, a sacrificial gelatin (catalogue number G6144-100G, Sigma) hydrogel solution of 12 mg ml^−1^ in phosphate-buffered saline (PBS; catalogue number 10010049, Thermo Fisher Scientific) was injected into the central area and solidified at −20 °C for 15 min. This step aims to minimize the generation of bubbles between different regions during the following cell loading process. Then, a mixture of HUVECs, hMSCs, Reh B-ALL cells and bone marrow mononuclear cells in fibrin solution (3 mg ml^−1^ in PBS) containing 2 U ml^−1^ thrombin (catalogue number 604980-100U, Sigma) was infused into the inner ring area and gelled at room temperature for 10 min. To recreate the endosteal niche, a mixture of NHLF and hMSC-derived osteoblast in fibrin solution (3 mg ml^−1^ in PBS) containing 2 U ml^−1^ thrombin was then loaded into the outer ring area by a gentle vacuum suction. Following the gelation, cell culture media was added into the four media reservoirs and the chip was incubated at 37 °C for 30 min, during which the gelled gelatin will become liquefied and be removed. Finally, HUVECs were seeded to cover the central area and establish interconnection with vessels formed by HUVECs in the inner ring area, which provided vascular openings at the central region. Since multiple types of cell were cultured within a single chip, cell culture media was chosen to mainly support the long-term maintenance of bone marrow immune cells and tissue structure with a mixture of SFEM-II (STEMCELL Technologies), EGM-2, RMPI1640 at a ratio of 1:2:1 and supplement with a cytokine cocktail (Thrombopoietin (TPO; catalogue number 300-18, Peprotech), IL-6 (catalogue number 200-06, Peprotech), IL-3 (catalogue number 200-03, Peprotech), FMS-like tyrosine kinase 3 ligand (Flt3-L; catalogue number 300-19, Peprotech) and stem cell factor (SCF; catalogue number 300-07, Peprotech), all at 12.5 ng ml^−1^) and recombinant human VEGF-165/VEGFA (catalogue number 230-00012-10, RayBiotech) at 10 ng ml^−1^. Fresh media was added to the four media reservoirs and the top of the device every day before old media was discarded or collected for future analysis and profiling of cytokine secretion. After 7-day culture, the as-prepared leukaemia chips were ready for characterization of CAR T cell functionality.

To test patient-specific CAR T cell response, we used patient-derived bone marrow mononuclear cells (PD7606, PD7813 and PD8012) to prepare patient-specific leukaemia chips (1 million cells per chip), and evaluated their own 4-1BBζ-CAR T cells produced from matched peripheral blood mononuclear cells (PD7607, PD7814 and PD8009) after 5-day (PD7813 and PD8012) or 9-day (PD7606) on-chip culture. To improve survival of patient-derived bone marrow mononuclear cells during on-chip culture, IL-7 (catalogue number 200-07, Peprotech) was added into cell culture media at 12.5 ng ml^−1^. These matched patient samples were provided by the UPENN Stem Cell and Xenograft Core of University of Pennsylvania School of Medicine (Research Resource Identifier (RRID) SCR_010035). Of note, the bone marrow stroma cells were from healthy donors owing to the unavailability of matched patient stromal samples.

### Scanning electron microscopy

Human leukaemia bone marrow chips were prepared and cultured for 7 days following the procedure described above. After being washed 3 times with PBS, the leukaemia chips were fixed with 2% glutaraldehyde in PBS (catalogue number 16010, Electron Microscopy Sciences) for 1 h. The fixed leukaemia chips were then manually cut into slices with a thickness of about 2 mm and dehydrated in a graded series of ethanol solutions. In brief, leukaemia chip samples were first dehydrated sequentially in 30%, 50%, 70%, 80% and 90% ethanol for 10 min and finally in 100% ethanol 3 times, each time for 20 min. The dehydrated leukaemia chip samples were then dried using a CO_2_ critical point dryer (Samdri-PVT-3D, Tousimis) according to the manufacturer’s instructions. The dried leukaemia chip samples were mounted on stubs and sputtered with gold palladium for 30 s (~12 nm coating), after which scanning electron microscopy (SEM) images were acquired using a Gemini 300 FESEM with Gatan 3View (Zeiss).

### Time-lapse live microscopy

To clearly visualize and distinguish T cells, leukaemia blasts and niche cells, we labelled CAR T cell or Mock T cell with Vybrant DiD cell-labelling solution (catalogue number V22887, Thermo Fisher Scientific) and used Reh leukaemia blasts and HUVECs that were genetically engineered to express with GFP (prepared in I. Aifantis’s lab or bought from Applied Biological Materials) or RFP (Angio-Proteomie), respectively. Also, K562-meso-19-mCherry- and VE-CAD-GFP-expressing HUVECs were used when evaluating 4-1BBζ-CAR and 4-1BBζ-CAR-IL18 under Nikon C2i confocal microscopy. We infused CAR T cells into the leukaemia chip via the central sinus and allowed the cells to interact for 2–4 days. After the defined incubation, the chip was mounted on an inverted microscope (Zeiss Axio Observer.Z1) with a motorized stage and an environment control incubation chamber (Okolab) to maintain 37 °C with 5% CO_2_. Phase contrast and fluorescence images were recorded every 5 min for 12 h using a digital CMOS camera (ORCA-Flash4.0 LT, Hamamatsu Photonics) with a 20× objective. In addition, single T cell migration was also monitored every 30 s in a time period of 4 h (in total, 481 frames). Cell motility parameters were assessed via tracking of single T cell (>100 cells per condition) in ImageJ (NIH) using Manual Tracking plug-in where the average cell migration speed was defined by the distance travelled in a unit time calculated using the corresponding coordinates at across frames. Of note, T cells can move out of the fixed field of view and thus the migration speed of T cells was quantified across the time frames when T cells were still present during the observation period.

### Modelling remission, relapse and resistance post CAR T cell therapy on-chip and off-chip

Clinical diagnosis of leukaemia considered leukaemia burden in bone marrow below 5% as a remission while 25% as relapse as non-response (relapse and resistance), although PCR or flow cytometry-based clinical characterization regarded a lower percentage (0.01–0.1%) of leukaemia burden as remission^46–49^. First, we calculated about 10,000 Reh B-ALL cells present in the leukaemia after 7-day culture with an initial seeding density at 1 × 10^6^ cells per ml (approximately 3,000 Reh B-ALL cells, day 0). Treatment with 10,000 CAR T cells (ratio of effector to tumour cell = 1:1) achieved remission in leukaemia chip. Thus, to replicate the resistance scenario, we lowered the ratio of effector to tumour cell to 1:4, such as 2,500 CAR T cells per chip compared with 10,000 in the remission scenario. To replicate relapse scenario, we spiked CD19^−^ leukaemia blasts (1% or 5%) into the whole leukaemia population when preparing leukaemia chips and estimated 150 of CD19^−^ leukaemia blasts per chip (5% condition). Following this set-up, we chronologically monitored the leukaemia burden (CD19^+^ leukaemia with GFP signal while CD19^−^ leukaemia with mCherry signal) over 14 days, as well as CAR T cell functionality. GFP-expressing and CD19-KO mCherry-expressing Reh B-ALL cell lines were generated following the protocol described in our previous study^29^. Leukaemia chips were built with different initial leukaemia burdens by tuning the initial leukaemia cell seeding density (that is, low burden 1 × 10^6^ cells per ml and high burden 4 × 10^6^ cells per ml). To model how a bridge chemotherapy can improve CAR T cell therapy response, the chip was treated with 20 nM VCR (catalogue number sc-201434, Sigma-Aldrich) for 24 h before administration of CAR T cell treatment, using protocol developed from our previous on-chip study of leukaemia stromal niche^28–30^.

Different response scenarios were also modelled with well-plate-based 2D co-culture. For example, a co-culture of 10,000 CAR T cells and 10,000 leukaemia blasts (GFP^+^ Reh) was prepared to approximate the remission condition and a co-culture of 2,500 CAR T cells and 10,000 leukaemia blasts to replicate the resistance condition, whereas a co-culture of 10,000 CAR T cells and 10,000 leukaemia blasts of which 5% are CD19^−^ (mCherry^+^ Reh) was to reproduce relapse condition. Following the set-up, leukaemia blasts were collected and quantified using a hemocytometer at different time points, days 1, 3, 5 and 7, under Zeiss Axio Observer.Z1 fluorescence microscopy.

### Collecting and processing clinical data

Clinical data of tumour burdens in patients with leukaemia during CAR T cell therapy at different time points were adopted and pooled from our recent study^50^. Previously, we collected information of 209 patients from different clinical studies where some studies reported clinical data of individual patients and others provided preprocessed clinical data with only statistical values such as medians. Therefore, we regarded statistical values of different groups (containing one or more individuals) of patients as representative individuals. In brief, publicly available figures from different clinical studies were digitalized with WebPlotDigitizer (https://automeris.io/WebPlotDigitizer) to collect data points that were then converted from peripheral blood to bone marrow if needed and grouped into 14 remission cases, 7 resistance cases and 11 relapse cases. To plot the curves of leukaemia burden of different response scenarios, nonlinear fitting was applied to remission and relapse responses and linear regression was applied to resistance response all with 95% confidence intervals using GraphPad Prism.

### Immunostaining

To characterize the cellularity of tissue-engineered bone marrow niche on-chip, leukaemia chips were stained with antibody targeting different cell populations, for example, vascular cells (FITC anti-human CD31, 1:50, catalogue number 303104, BioLegend), bone marrow MSCs (APC anti-human CD90/Thy1, 1:50, catalogue number 328113, BioLegend), hematopoietic cells (APC anti-human CD45, 1:50, catalogue number 304012, BioLegend), T cells (CD3, FITC anti-human CD3, 1:20, catalogue number 317306, BioLegend, PE anti-human CD3, 1:20, catalogue number 317308, BioLegend, and APC anti-human CD3, 1:20, catalogue number 300412, BioLegend; CD4, PE anti-human CD4, 1:20, catalogue number 317410, BioLegend; CD8, FITC anti-human CD8a, 1:20, catalogue number 300906, BioLegend, PE anti-human CD8a, 1:20, catalogue number 300908, BioLegend, APC anti-human CD8a, 1:20, catalogue number 300912, BioLegend), monocytes (Alexa Fluor 647 anti-human CD14, 1:50, catalogue number 325611, BioLegend) and macrophages (Alexa Fluor 488 anti-human CD68, 1:50, catalogue number 333812, BioLegend), and hematopoietic stem cells (APC anti-human CD34, 1:50, catalogue number 343510, BioLegend).

To quantify the deposition of ECMs during the 7-day culture, leukaemia chips were fixed with fixation buffer (catalogue number 420801, BioLegend) each day and stained with DyLight488-Laminin (catalogue number PA5-22901, Thermo Fisher Scientific), PE-fibronectin (catalogue number IC1918P, R&D Systems) and Alexa Fluor 647-Collagen IV (catalogue number 51-9871-80, Thermo Fisher Scientific) all at a dilution of 1:50.

To quantify T cell activation on-chip, leukaemia chips were first incubated with FITC anti-human CD3 (1:20, catalogue number 317306, BioLegend), PE anti-human CD69 (1:50, catalogue number 310906, BioLegend) or PE anti-human CD25 (1:50, catalogue number 302606, BioLegend), as well as Human TruStain FcX (1:50, catalogue number 422301, BioLegend) for block of Fc receptor for about 4 h at 4 °C, then thoroughly washed 3 times with PBS followed by fixation using FluoroFix Buffer (catalogue number 422101, BioLegend) for 20 min. To stain Ki67 (1:50, APC anti-human Ki67, catalogue number 350514, or Alexa Fluor 488 anti-human Ki67, catalogue number 350508, BioLegend), leukaemia chips were first permeabilized with 0.3% Triton X-100 (catalogue number 11332481001, Sigma-Aldrich) for 20 min and then thoroughly washed 3 times after incubation with antibodies at a dilution of 1:50 for 4 h at room temperature or overnight at 4 °C. To indicate CAR T cell, leukaemia chips were first incubated with biotinylated monoclonal anti-FMC63 scFv (CAR) antibody (1:50, catalogue number FM3-BY54, ACRO Biosystems), followed by incubation with PE (catalogue number 405245, BioLegend) or APC (catalogue number 405243, BioLegend) conjugated streptavidin (1:50), or directly stained with FITC-labelled monoclonal anti-FMC63 scFv antibody (1:50, catalogue number FM3-FY45P1, ACRO Biosystems) or APC-labelled monoclonal anti-FMC63 scFv antibody (1:50, catalogue number FM3-AY54P1, ACRO Biosystems). The leukaemia chips were finally incubated for 10 min with 4′,6-diamidino-2-phenylindole (DAPI; catalogue number D1306, Thermo Fisher Scientific) to counterstain nuclei.

To quantify the activation of bone marrow niche cells, Alexa Fluor 647 anti-human CD14 (1:50, catalogue number 325612, BioLegend) and PE anti-human HLA-DR (1:50, catalogue number 307606, BioLegend) were used to stain HLA-DR expression on CD14^+^ monocytic cells, whereas Alexa Fluor 488 anti-human CD54 (1:50, ICAM-1, catalogue number 353129, BioLegend) and PE anti-human CD31 (1:50, catalogue number 303106, BioLegend) or PE anti-human VE-cadherin (1:50, catalogue number 348506, BioLegend) were used to characterize expression of ICAM-1 on vascular cells.

All the stained samples were kept in Cell Staining Buffer (catalogue number 420201, BioLegend) and imaged with a Nikon C2i confocal microscope unless stated otherwise. The obtained images were processed in Nikon NIS-Elements Microscope Imaging Software (Ar version) and mean intensity of each maker was quantified using ImageJ (NIH) or Fiji (version 2).

### Flow cytometry

T cell activation marker was detected using FITC anti-human CD3 (1:20), PE anti-human CD69 (1:50), PE anti-human CD25 (1:50) and Human TruStain FcX (1:50). Single-cell suspensions of leukaemia chip samples (CAR and Mock) were first prepared by off-chip recovery with nattokinase (50 Fu ml^−1^, NSK-SD, Pure Encapsulations)^28–30^, and then respectively incubated with antibodies for 30 min at 4 °C. Cells were washed three times and stained with LIVE/DEAD Fixable Aqua Dead Cell Stain Kit (1:1,500, catalogue number L34957 Thermo Fisher Scientific) to mark live/dead cells. All samples were analysed via an LSRII UV flow cytometer (BD Biosciences) by FACSDiva (version 5) and processed via FlowJo version 10 (Treestar, BD Biosciences). Fluorescence compensations were prepared by incubating respective antibodies with CompBead Anti-Mouse Ig, κ/Negative Control Particles Set (catalogue number 552843, BD Biosciences). All T cells were gated as live CD3^+^ population (Extended Data Fig. 3h).

### Profiling and quantification of cytokine secretion

Qualitative profiles of cytokine secretion from leukaemia chips were examined by using a Human Inflammation Array C3 membrane kit (catalogue number AAH-INF-3-8, RayBiotech) according to the manufacturer’s protocols. In brief, supernatants were collected from 2–4 leukaemia chips after on-chip infusion of CAR T cell and Mock T cell for certain time points, centrifuged at 2,000 × *g* for 20 min at 4 °C to remove cellular debris, and then incubated overnight with Human Inflammation Array membranes (or stored at −20 °C for future assays). Biotinylated antibody cocktail was incubated with the membranes at 4 °C overnight, followed by washing 3 times and incubation with HRP-labelled streptavidin at 4 °C overnight. The mixture of Detection Buffer C and D was then applied for 2 min to visualize chemiluminescence at room temperature. Imaging was obtained by using a ChemiDoc imaging system (Bio-Rad). The mean intensity of each spot was quantified in ImageJ (NIH) or Fiji (version 2) using Protein Array Analyzer plug-in (written by G. Carpentier, Faculté des Sciences et Technologies, Université Paris).

Absolute concentrations of different cytokines were measured with respective enzyme-linked immunosorbent assay (ELISA) kits, such as Human IFNγ ELISA MAX Deluxe (catalogue number 430104, BioLegend), Human IL-10 ELISA MAX Deluxe (catalogue number 430604, BioLegend), Human IL-13 Uncoated ELISA Kit (catalogue number 88-7439-22, Thermo Fisher Scientific), Human Perforin ELISA Set (catalogue number ab83709, Abcam), Human Granzyme B Set (catalogue number DY2906-05, R&D Systems), Human CCL1/I-309 DuoSet (catalogue number DY272, R&D Systems) and MIP-1β Human Instant ELISA Kit (catalogue number BMS2030INST, Invitrogen), according to the manufacturer’s protocols.

### scRNA-seq analysis

The leukaemia samples (designated as HD CAR, PD CAR, Mock and None) were engineered and cultured on-chip for 7 days as described above, followed by 2-day treatment with HD CAR T cell, PD CAR T cell, Mock T cell or left untreated. Single-cell suspensions of leukaemia samples were prepared by off-chip recovery with nattokinase, pre-labelled with different anti-human hashtag antibodies (TotalSeq, BioLegend), that is, Hashtag1-GTCAACTCTTTAGCG, catalogue number 394601; Hashtag2-TGATGGCCTATTGGG, catalogue number 394603; Hashtag3-TTCCGCCTCTCTTTG, catalogue number 394605; Hashtag4-AGTAAGTTCAGCGTA, catalogue number 394607, all at a 1:250 dilution and mixed into one sample. Correspondingly, freshly thawed HD CAR T cell, PD CAR T cell, Mock T cell and bone marrow mononuclear cells were pre-labelled with different anti-human hashtag antibodies, mixed into one sample and used controls. The libraries were prepared using the Chromium Single Cell 3′ Reagent Kits (v3): Single Cell 3′ Library & Gel Bead Kit v3 (PN-1000075), Single Cell 3′ Chip Kit v3 (PN-1000073) and i7 Multiplex Kit (PN-120262) (10x Genomics) and following the Single Cell 3′ Reagent Kits (v3) User Guide (manual part number CG000183 Rev B). Libraries were then run on an Illumina NovaSeq 6000 using 28 bp read 1, 8 bp i7 index and 91 bp read 2.

The raw sequencing results from the 10x Genomics platform were processed using CellRanger (version 3.1). In brief, CellRanger was used to generate a count matrix by aligning output reads, filtering the empty dropouts and counting unique molecular identifiers. We used Ensemble hg38/GRCh38 as the reference genome for read alignment. Further analyses, including quality control and data filtering, the identification of highly variable genes, dimensionality reduction, standard unsupervised clustering algorithms and the discovery of differentially expressed genes (DEGs), were performed using the R package Seurat (version 4.1.1)^81^.

To visualize the data, the dimensionality of the scaled integrated data matrix was reduced to project the cells in 2D space using principal component analysis followed by uniform manifold approximation and projection (UMAP) (https://umap-learn.readthedocs.io/) based on 40 PCs with 30 nearest neighbours used to define the local neighbourhood size with a minimum distance of 0.3 for the datasets^82,83^. The resulting PCs were also used as a basis for partitioning the dataset into clusters using a smart local moving community detection algorithm^84^ with 30 nearest neighbours for the datasets. A range of resolutions (0.1–10) was utilized to establish a sufficient number of clusters to separate known populations based on the expression of established markers. Cell clusters were annotated based on DEGs and published marker genes for cell types. To find markers that define individual clusters, we performed pairwise differential expression analysis using the Wilcoxon rank sum test with Bonferroni multiple-comparison correction for each cluster against all other clusters for genes that were detected in at least 10% of the cluster cells, keeping the genes that were significant in each of the comparisons (fold-change difference >10% with adjusted *P* < 0.01).

### Statistics and reproducibility

All the results, including error bars in the graphs, are shown as mean and standard error of the mean (s.e.m.). The biological and technical replicates and repetitions for each experiment are listed in the respective figure legends. Representative images shown in each figure are from one of the three technical replicates with similar results. A significant difference between two groups was determined by unpaired two-tailed Student’s *t*-test with GraphPad Prism software (version 10), as indicated in the figure legends. Multiple groups were compared by one-way or two-way analysis of variance (ANOVA) as indicated in the figure legends.

### Reporting summary

Further information on research design is available in the Nature Portfolio Reporting Summary linked to this article.

## Supplementary information

Supplementary Information Supplementary Figs. 1–9 and captions of Supplementary Videos 1–5.

Reporting Summary

Supplementary Video 1 2D time-lapse imaging showed CAR T cell extravasation, related to Extended Data Fig. 3a. T cells are in red, Reh B-ALL leukaemia blasts are in green, and vessels (HUVECs) are in blue.

Supplementary Video 2 2D time-lapse imaging showed CAR T cell infiltration, related to Extended Data Fig. 3b. T cells are in red, Reh B-ALL leukaemia blasts are in green, and vessels (HUVECs) are in blue.

Supplementary Video 3 2D time-lapse imaging showed the process of killing a Reh B-ALL blast (green) by a CAR T cell (red), related to Extended Data Fig. 3c.

Supplementary Video 4 Confocal time-lapse imaging showed the process of extravasation, infiltration and killing of healthy donor-derived CAR T cell in the leukaemia bone marrow chip. Vessel was formed with VE-CAD-GFP-expressing HUVECs (in green). Leukaemia blast was K562-meso-19-mCherry (in yellow). CAR T cells were stained with DiD dye (in red). The video was captured at 5 min per frame within 14 h using Nikon C2i confocal microscopy and a 20× objective.

Supplementary Video 5 Confocal time-lapse imaging showed the process of extravasation, infiltration and killing of patient-derived CAR T cell in the leukaemia bone marrow chip. Vessel was formed with VE-CAD-GFP-expressing HUVECs (in green). Leukaemia blast was K562-meso-19-mCherry (in yellow). CAR T cells were stained with DiD dye (in red). The video was captured at 5 min per frame within 14 h using Nikon C2i confocal microscopy and a 20× objective.

Supplementary Data 1 Statistical source data.

Supplementary Data 2 Statistical source data.

Supplementary Data 3 Statistical source data.

Supplementary Data 4 Statistical source data.

Supplementary Data 5 Statistical source data.

Supplementary Data 6 Statistical source data.

## Source data

Source Data Fig. 1 Statistical source data.

Source Data Fig. 2 Statistical source data.

Source Data Fig. 3 Statistical source data.

Source Data Fig. 4 Statistical source data.

Source Data Fig. 5 Statistical source data.

Source Data Fig. 6 Statistical source data.

Source Data Extended Data Fig. 2 Statistical source data.

Source Data Extended Data Fig. 3 Statistical source data.

Source Data Extended Data Fig. 4 Statistical source data.

Source Data Extended Data Fig. 5 Statistical source data.

Source Data Extended Data Fig. 6 Statistical source data.

Source Data Extended Data Fig. 7 Statistical source data.

Source Data Extended Data Fig. 8 Statistical source data.

## Data Availability

The data supporting the results in this study are available within the paper and its Supplementary Information. The scRNA-seq data are available in the Gene Expression Omnibus (GEO) under accession number GSE293390. The Ensemble hg38/GRCh38 reference genome for scRNA-seq read alignment is available on NCBI Datasets (GCF_000001405.40). The raw and analysed datasets generated during the study are available from the corresponding authors on reasonable request. Source data are provided with this paper.
