# Supplementary material for: Bioengineered immunocompetent preclinical trial-on-chip tool enables screening of CAR T cell therapy for leukaemia
Source: Nat Biomed Eng. 2025 Jul 1;9(12):2098–114. doi: 10.1038/s41551-025-01428-2 (PMC12705464; doi:10.1038/s41551-025-01428-2)
Supplement: Supplementary file 1 — Supplementary Figs. 1–9 and captions of Supplementary Videos 1–5. [file 41551_2025_1428_MOESM1_ESM.pdf]

nature biomedical engineering

Supplementary information https://doi.org/10.1038/s41551-025-01428-2

Bioengineered immunocompetent preclinical trial-on-chip tool enables screening of CAR T cell therapy for leukaemia

In the format provided by the authors and unedited

Table of Contents

Supplementary Fig. S1..2

Supplementary Fig. S2..3

Supplementary Fig. S3.. 4

Supplementary Fig. S4..6

Supplementary Fig. S5..7

Supplementary Fig. S6..8

Supplementary Fig. S7..10

Supplementary Fig. S8..12

Supplementary Fig. S9..13

Supplementary Video 1-5...15

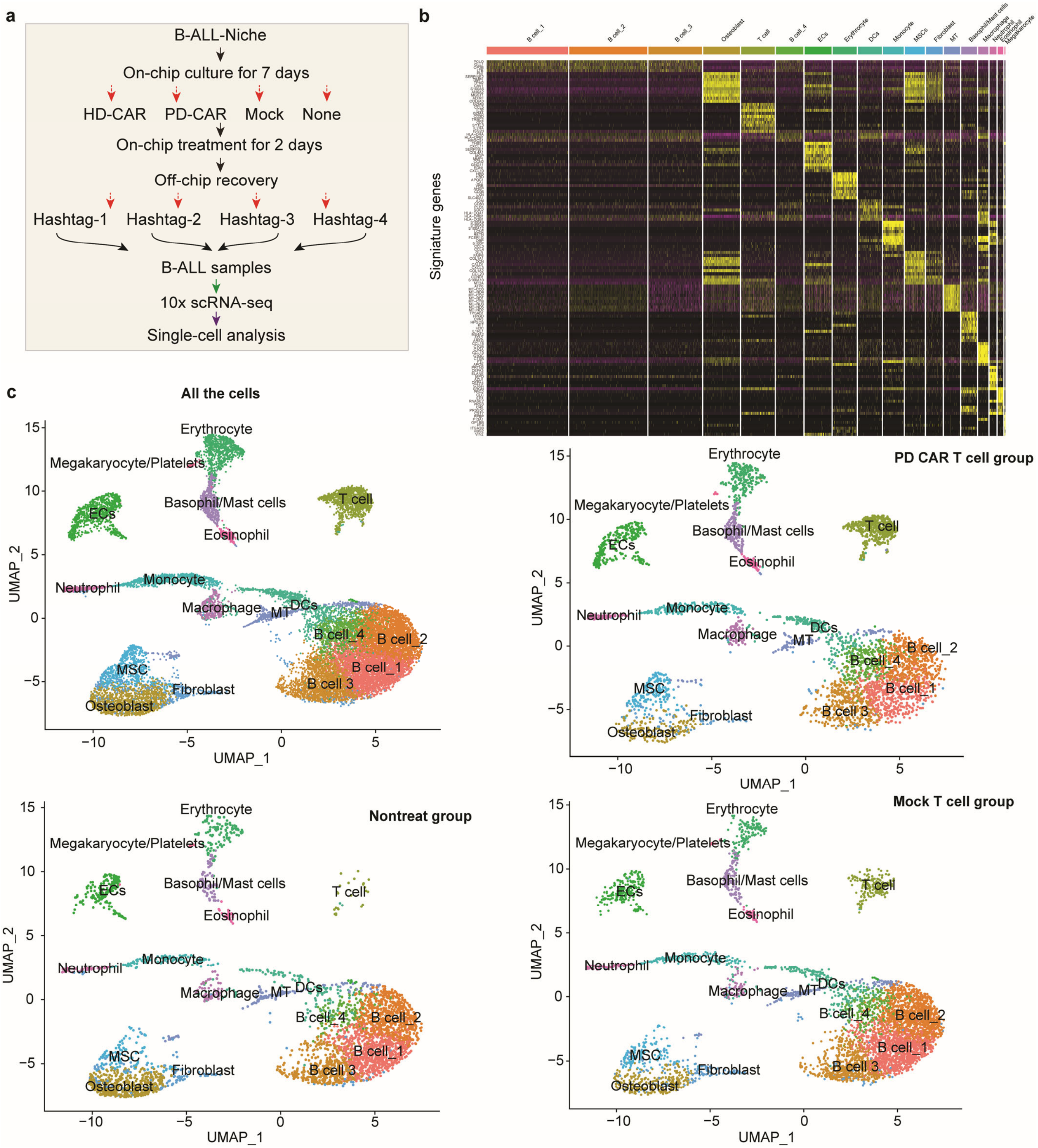

Supplementary Fig. S1. scRNA-seq mapping of engineered bone marrow niche. (a) The schematic showing the sample preparation for scRNA-seq. (b) Signature genes (top 20) of each cluster identified. (c) The UMAP presentation of different cell populations from different samples, where bone marrow niche chips were treated with HD CAR T cell, PD CAR T cell, Mock T cell, or left non-treated, corresponding to **Fig. 1-3**.

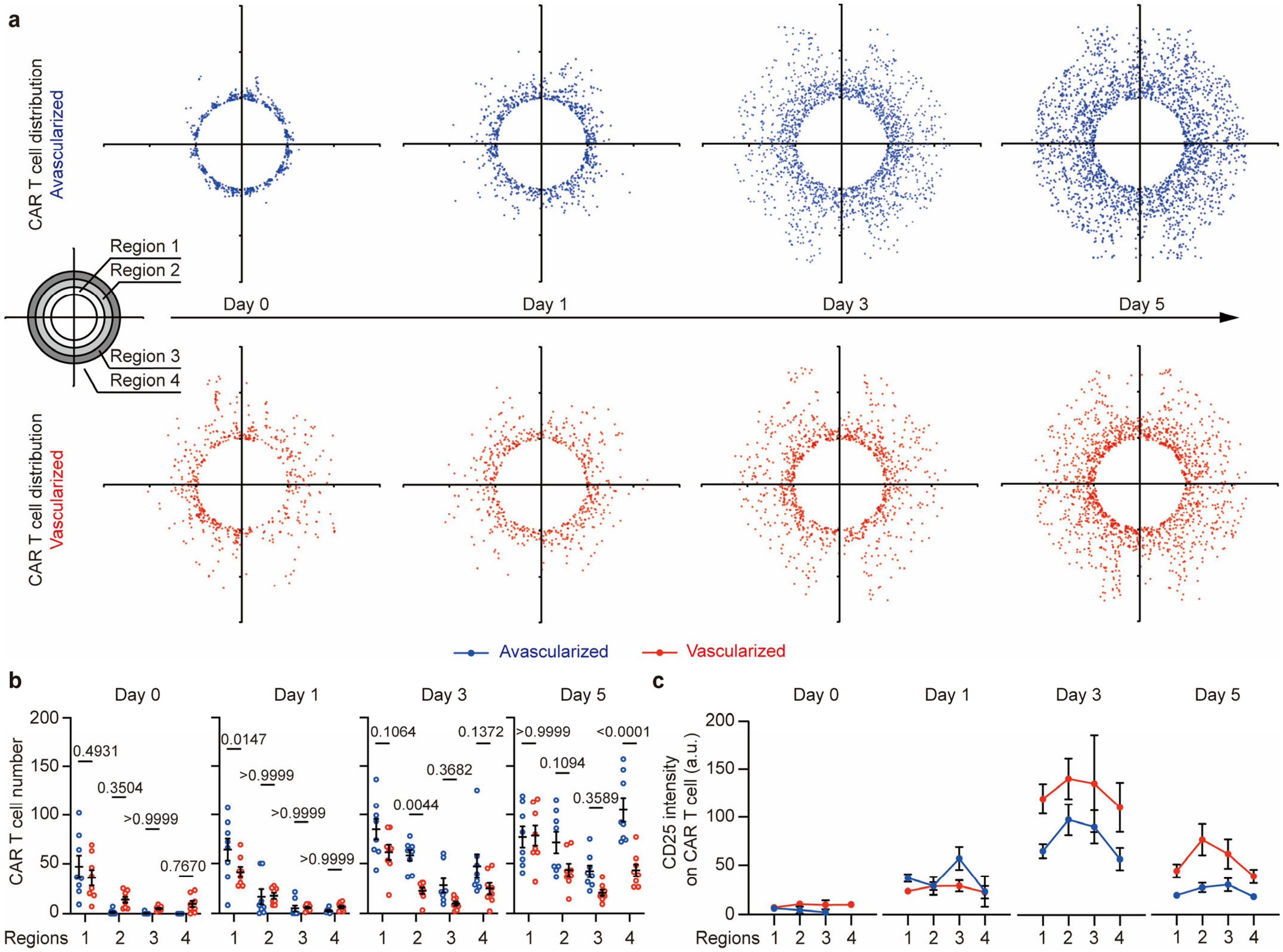

Supplementary Fig. S2. CAR T cell distribution and activation in vascularized and avascularized leukemia chips. (**a**) CAR T cells migrated into medullary cavity and endosteal regions at different time points day 0, 1, 3, and 5 were marked. Each data represents a CAR T cell. The center of the leukemia device is set as the origin. (**b**) The number of CAR T cells across different regions (Region 1: 1000-1250μm; Region 2: 1250-1500μm; Region 3: 1500-1750μm; Region 4: >1750μm) in the leukemia devices. Two-way ANOVA followed by Tukey's post hoc test, mean and s.e.m. (**c**) Quantification of T cell activation with surface expression of CD25 on CAR T cells across 4 regions. Data for each graph was collected and pooled from 8 devices (n=8) with CAR T cells from two healthy donors. Data are present as mean +/- s.e.m.

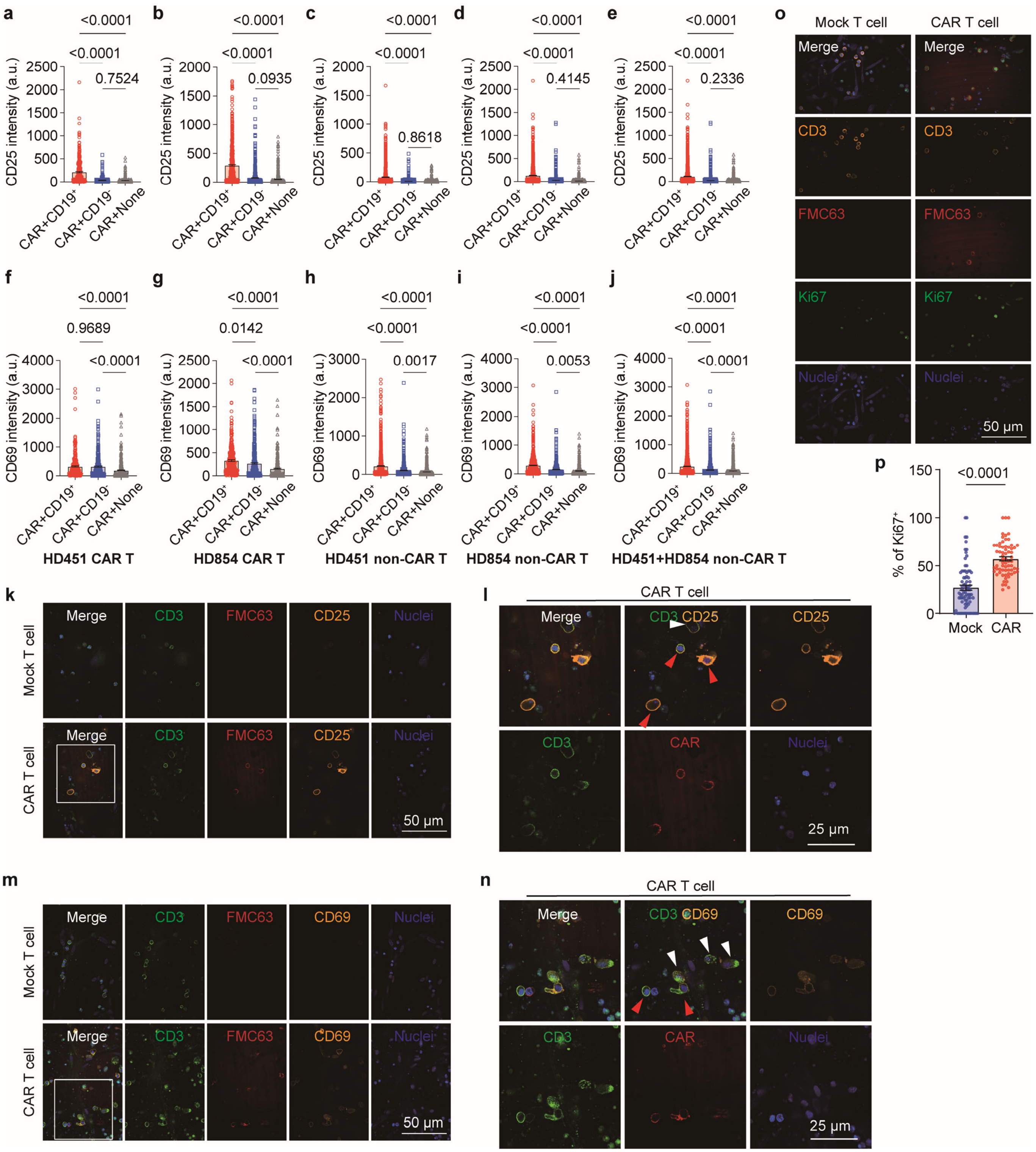

Supplementary Fig. S3. Activation of bystander T cells during CAR T cell activation on-chip. (**a-e**) Quantification of T cell activation with surface expression of CD25 on all $CD3^+$ T cells from CAR T cell treated leukemia chips (dose of 10,000 CAR T cells) established with CD19-expreesing leukemia ($CAR+CD19^+$), non-CD19 expressing leukemia on-chip ($CAR+CD19^-$), or no leukemia (CAR+None). Surface expression of CD25 on CAR T cells from healthy donor HD451 (**a**) and HD854 (**b**), and surface expression of CD25 on non-CAR expressing T cells from HD451 (**c**) and HD854 (**d**), and combined data (**e**). (**f-j**) Quantification of T cell activation with surface expression of CD69 on all $CD3^+$ T cells from

CAR T cell treated group. Surface expression of CD69 on CAR T cells from healthy donor HD451 (**f**) and HD854 (**g**), and surface expression of CD69 on non-CAR expressing T cells from HD451 (**h**) and HD854 (**i**), and combined data (**j**). Data in **a-j** was collected from three independent experiments (n=3). One-way analysis of variance (ANOVA) followed by Tukey's post hoc test, mean and s.e.m. (**k-n**) Representative images showing T cell activation with surface expression of (**k**,**l**) CD25 and (**m**,**n**) CD69 in all $CD3^+$ T cell from CAR T cell treated group (CAR) and Mock T cell treated group (Mock). (**l**,**n**) are enlarge of the insets from (**k**,**m**) CAR-expressing T cell (CAR-only, $CD3^+FMC63^+$) and non-CAR expressing T cell (non-CAR, $CD3^+FMC63^-$). Representative images were from one of three technical replicates (n=3) with similar results. (**o**,**p**) Ki67 in all $CD3^+$ T cell from CAR T cell treated group (CAR, left) and Mock T cell treated group (Mock, right). Data was collected from four technical replicates (n=4). Unpaired, two-sided, Student's t-test, mean and s.e.m.

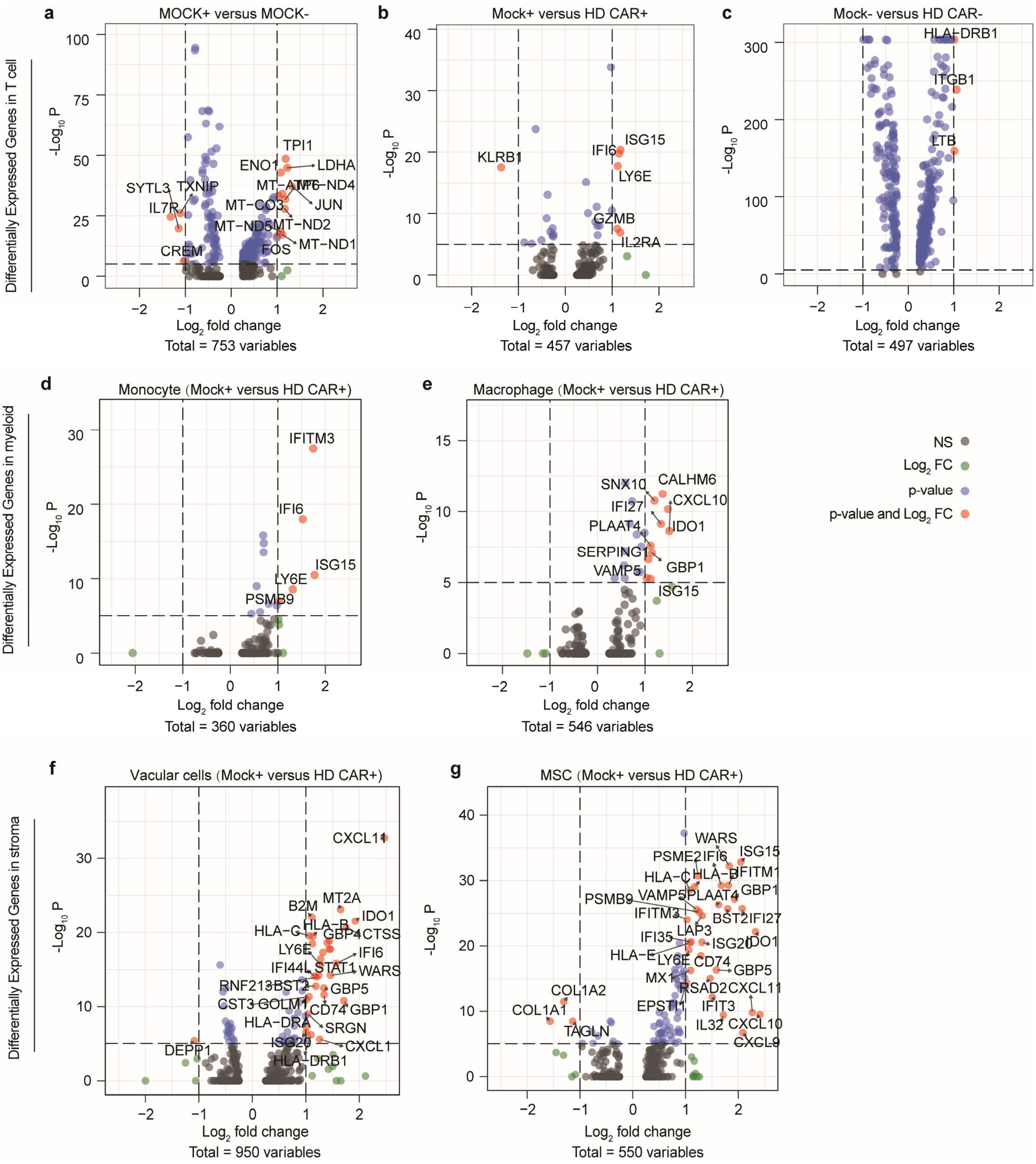

Supplementary Fig. S4. scRNA-seq mapping of leukemia chips treated with healthy donor (HD) derived CAR T cells. (**a**) Analysis of Differentially Expressed Genes (DEG) in Mock T cells before (Mock-) and after (Mock+) interaction with leukemia blasts on-chip for 2 days. (**b**,**c**) DEG analysis between CAR T cell (HD CAR) and Mock T cell after (**b**) and before (**c**) interaction with leukemia blasts on-chip for 2 days. (**d-g**) DEG analysis of monocyte (**d**), macrophage (**e**), vascular cells (**f**), and hMSC stromal cells (**g**) from chips treated with CAR T cell (HD CAR+) or Mock T cell (Mock+) for 2 days. Two-sided Wilcoxon Rank Sum test.

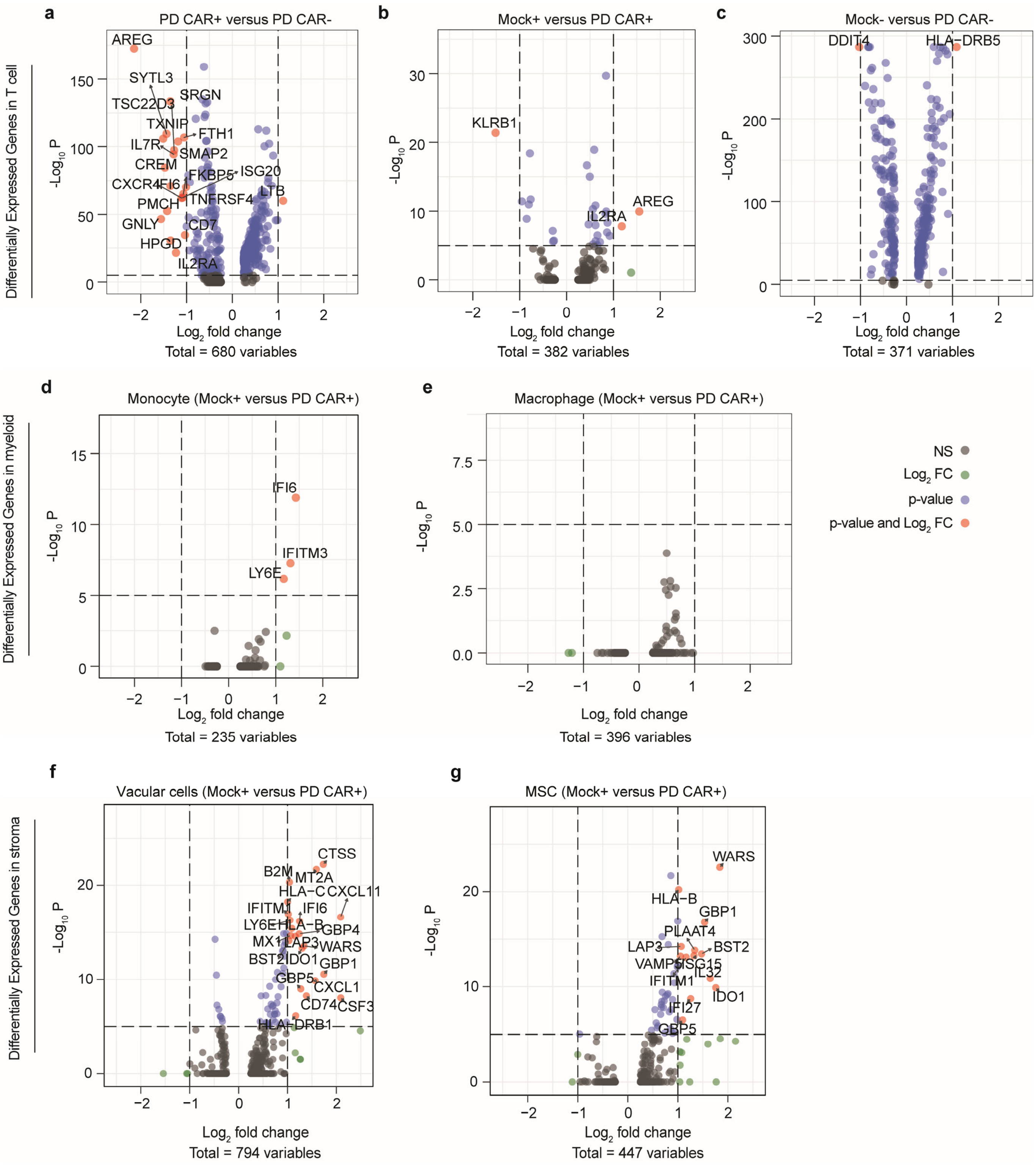

Supplementary Fig. S5. scRNA-seq mapping of leukemia chip treated with patient derived (PD) CAR T cell. (**a**) Analysis of Differentially Expressed Genes (DEG) in Mock T cells before (Mock-) and after (Mock+) interaction with leukemia blasts on-chip for 2 days. (**b**,**c**) DEG analysis between CAR T cell (PD) and Mock T cell after (**b**) and before (**c**) interaction with leukemia blasts on-chip for 2 days. (**d**-**g**) DEG analysis of monocyte (**d**), macrophage (**e**), vascular cells (**f**), and hMSC stromal cells (**g**) from leukemia chips treated with CAR T cell (PD CAR+) or Mock T cell (Mock+) for 2 days. Two-sided Wilcoxon Rank Sum test.

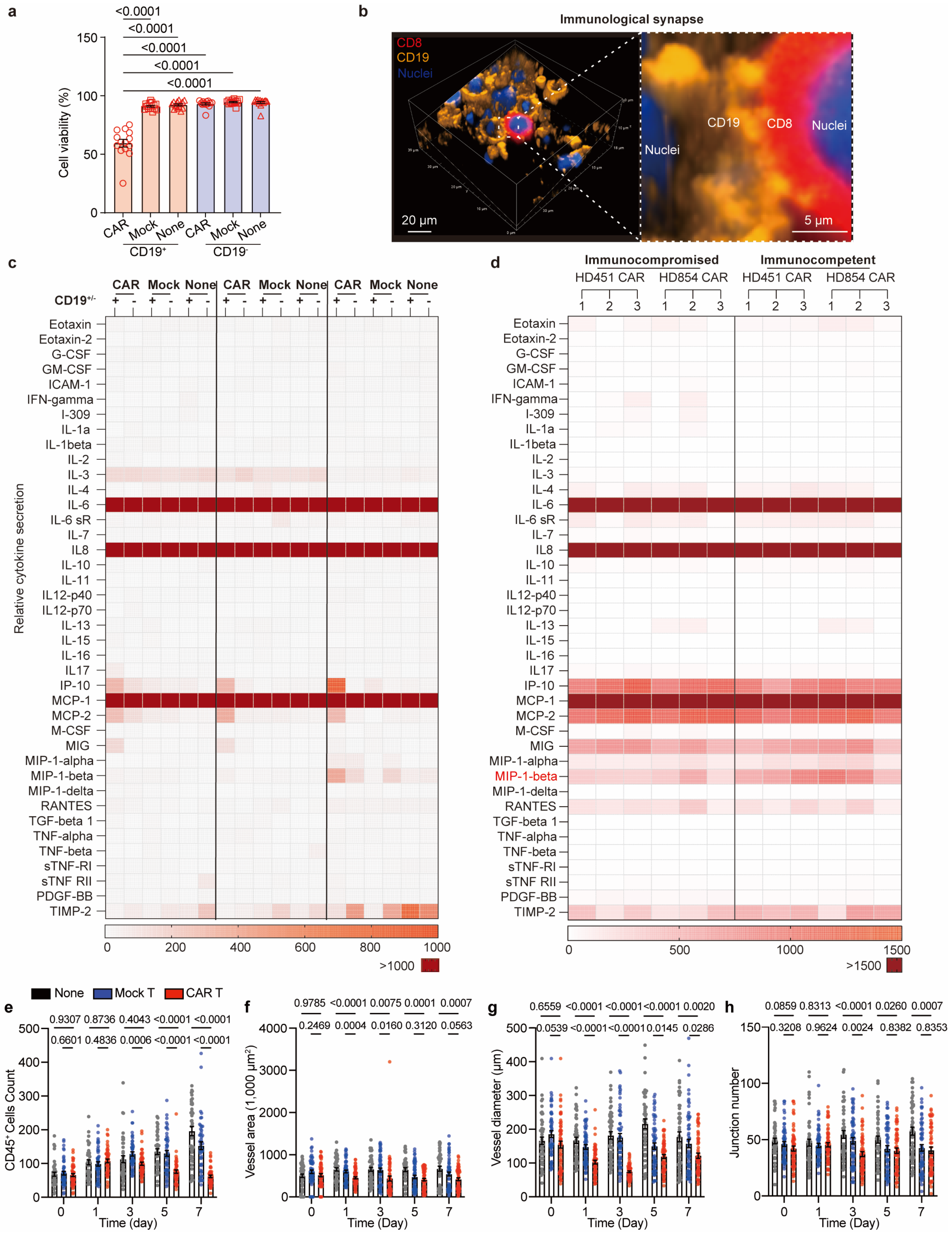
a
Cell viability (%)
CAR
Mock
None
CD19⁺
CD19⁻
<0.0001
b
Immunological synapse
CD8
CD19
Nuclei
20 μm
5 μm
c
CAR Mock None
CD19+/-
Relative cytokine secretion
>1000
d
Immunocompromised
Immunocompetent
HD451 CAR
HD854 CAR
>1500
Eotaxin
Eotaxin-2
G-CSF
GM-CSF
ICAM-1
IFN-gamma
I-309
IL-1a
IL-1beta
IL-2
IL-3
IL-4
IL-6
IL-6 sR
IL-7
IL8
IL-10
IL-11
IL12-p40
IL12-p70
IL-13
IL-15
IL-16
IL17
IP-10
MCP-1
MCP-2
M-CSF
MIG
MIP-1-alpha
MIP-1-beta
MIP-1-delta
RANTES
TGF-beta 1
TNF-alpha
TNF-beta
sTNF-RI
sTNF RII
PDGF-BB
TIMP-2
e
None
Mock T
CAR T
CD45⁺ Cells Count
Time (day)
f
Vessel area (1,000 μm²)
Time (Day)
g
Vessel diameter (μm)
h
Junction number

Supplementary Fig. S6. CAR T cell activation triggers systematic immune response from leukemia bone marrow niche. (**a**) Cell viability of leukemia bone marrow chips built with $CD19^+$ and $CD19^-$ leukemia blasts, which were respectively treated with 10,000 CAR T cell (CAR), Mock T cell (Mock), or left non-treated (None). Data was collected from three independent experiments (n=3). Unpaired, two-sided, Student's t-test, mean and s.e.m. (**b**) A representative 3D image showing the intercellular contact between CAR T cell (CD8 in red) and leukemia blasts (CD19 in yellow) where intercellular synapse is formed and cytolytic granules are released. (**c**) Profiling of cytokine secretion from leukemia chips that were established with $CD19^+$ (pos) and $CD19^-$ (neg) leukemia blast and treated with CAR T cell (CAR), Mock T cell (Mock) or left untreated (None). (**d**) Profiling of cytokine secretion from immunocompetent and immunocompromised leukemia chips that were established with or without bone marrow mononuclear cells and treated with two healthy CAR T cells products. Cytokine secretion profiles in c,d were examined from chips at day 2 by using a Human Inflammation Array C3 membrane kit, and data was collected from three independent experiments (n=3). (**e**) Count of $CD45^+$ cells on chips treated either with 10,000 of CAR T cells (CAR T, red), Mock T cells (Mock, blue) per chip for 7 days or left untreated (None, black). (**f**-**h**) Vascular network, where its vessel area (**f**), vessel diameter (**g**) and number of tight junction (**h**) were decreased at day 3 and recovered slightly in the following days when treated with CAR T cell, compared to those of leukemia devices either left untreated or treated with Mock T cell. Data in **e**-**h** was collected from three independent experiments (n=3), mean and s.e.m. Statistical difference was analyzed using a mixed model with Dunnett's multiple comparisons test.

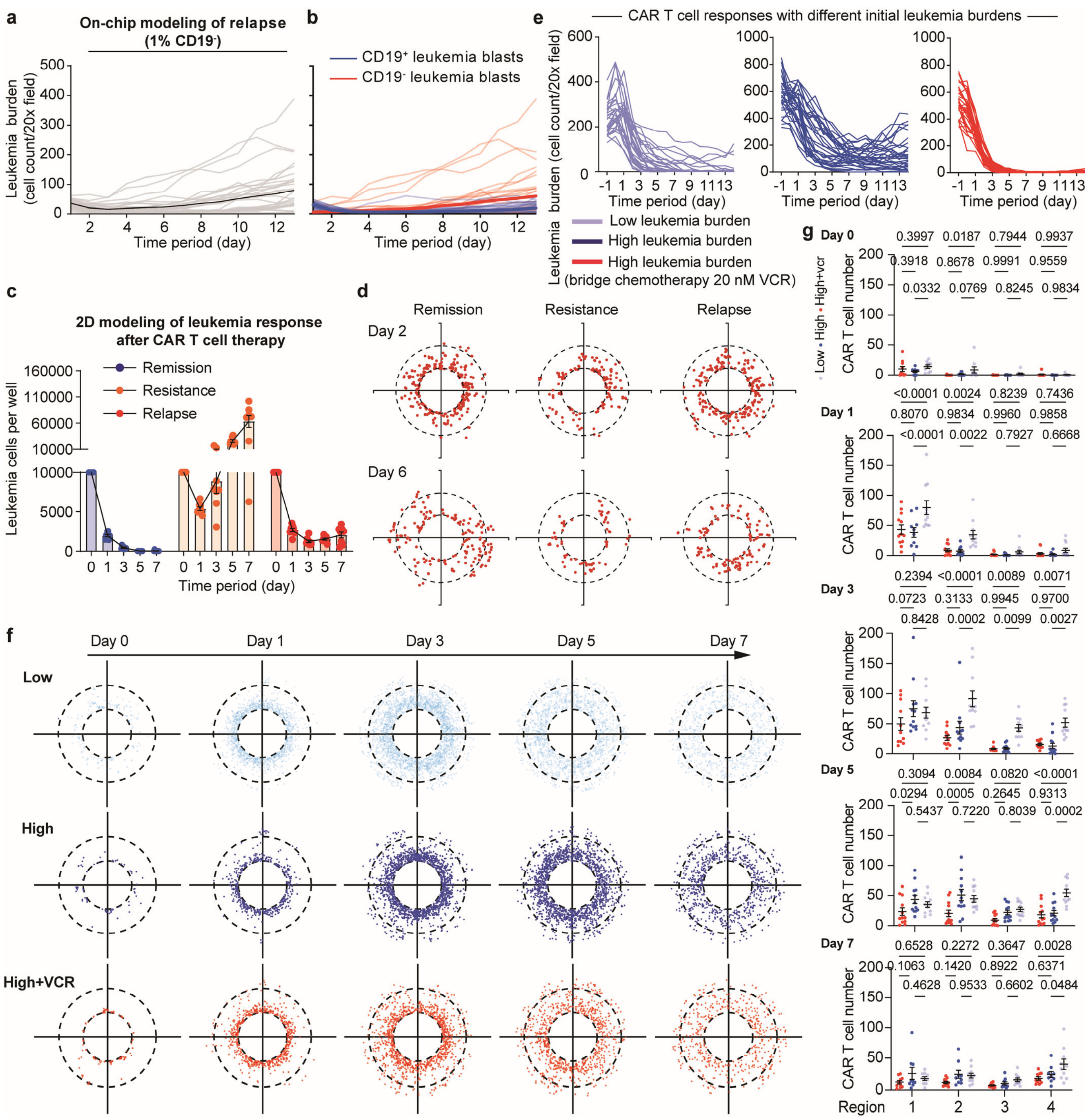

Supplementary Fig. S7. Modeling leukemia relapse post CAR T cell therapy. (**a**) Leukemia burden under CAR T cell therapy in the leukemia bone marrow niche spiked with 1% CD19$^-$ leukemia blasts. Data was collected from three independent experiments (n=3) each with 2-3 chips, where each chip has four random fields quantified (n=32 images). (**b**) The counts of CD19$^+$ (blue lines) and CD19$^-$ (red lines) leukemia blasts in the leukemia bone marrow niche spiked with 1% CD19$^-$ leukemia blasts, corresponding to (**a**). Data was collected from three independent experiments (n=3). (**c**) 2D modeling of leukemia response scenarios (i.e., remission, resistance, and relapse), corresponding to **Fig. 4a**. Data was collected from 4 independent experiments (n=4), mean and s.e.m. (**d**) Dynamic distribution of CAR T cells in remission, resistance, and relapse scenarios on day 2 and 6, corresponding to **Fig. 4c**. Each dot represents a CAR T cell. Black dash circles indicated the three concentric regions, central sinus, medullary cavity,

and endosteum. Representative data was from one of three technical replicates with similar results (n=3). (**e**) On-chip responses curve of CAR T cell therapy and (**f**) Dynamic distribution of CAR T cells in leukemia chips built with different initial leukemia burdens (i.e., low burden 1×10^6 cells/mL and high burden 4×10^6 cells/mL) and bridge chemotherapy (24-hour treatment of 20 nM vincristine, VCR). Data was collected from eight technical replicates (n=8), mean and s.e.m. (**g**) Quantification of distribution of CAR T cells in leukemia chips built with different initial leukemia burdens across 4 regions (Region 1: 1000-1250μm; Region 2: 1250-1500μm; Region 3: 1500-1750μm; Region 4: >1750μm, where the center of the leukemia device is set as the origin, 0μm). Two-way ANOVA followed by Tukey's post hoc test, mean and s.e.m. Data for each graph was collected and pooled from 8 devices (n=8) with CAR T cells from two healthy donors (n=2), corresponding to **Fig. 4g**.

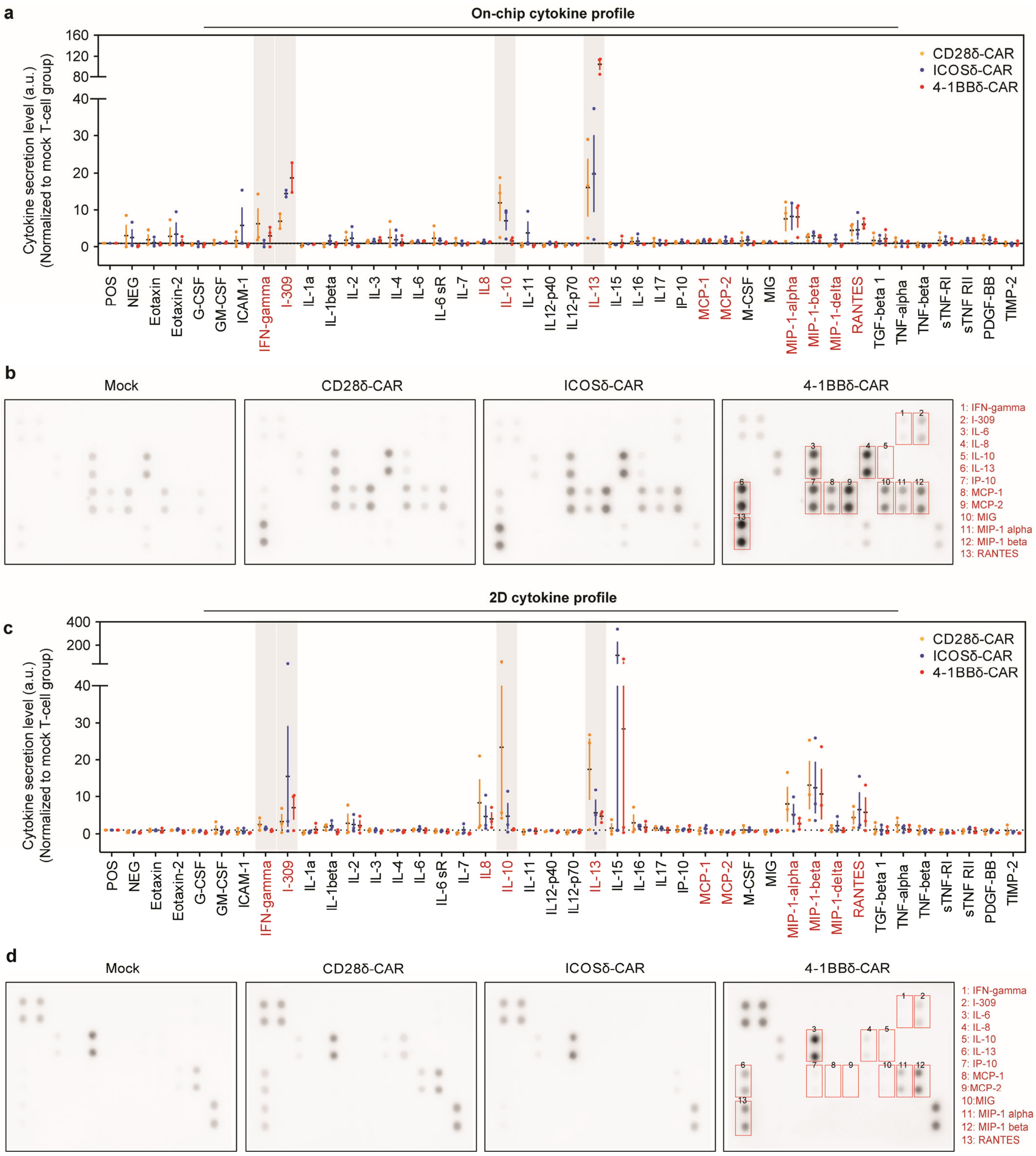

Supplementary Fig. S8. Cytokine secretion profiling of 2nd-gen CAR T cell products. Cytokine secretion profiles of Mock T cell, 2nd-gen CD28ζ-CAR, ICOSζ-CAR, and 4-1BBζ-CAR from either (**a**,**b**) on-chip or (**c**,**d**) 2D co-culture conditions at day 2 were examined by using a Human Inflammation Array C3 membrane kit. Cytokine secretion data in **a**,**c** from CAR T cell groups was normalized to that of Mock T cell group (absolute unit, a.u.). Data was collected from three biological replicates (n=3), i.e., CAR T-cell products from healthy donors ND164, ND365, and TMP497. Data are present as mean and s.e.m. Representative raw images in **b**,**d** were from three biological replicates with similar results (n=3).

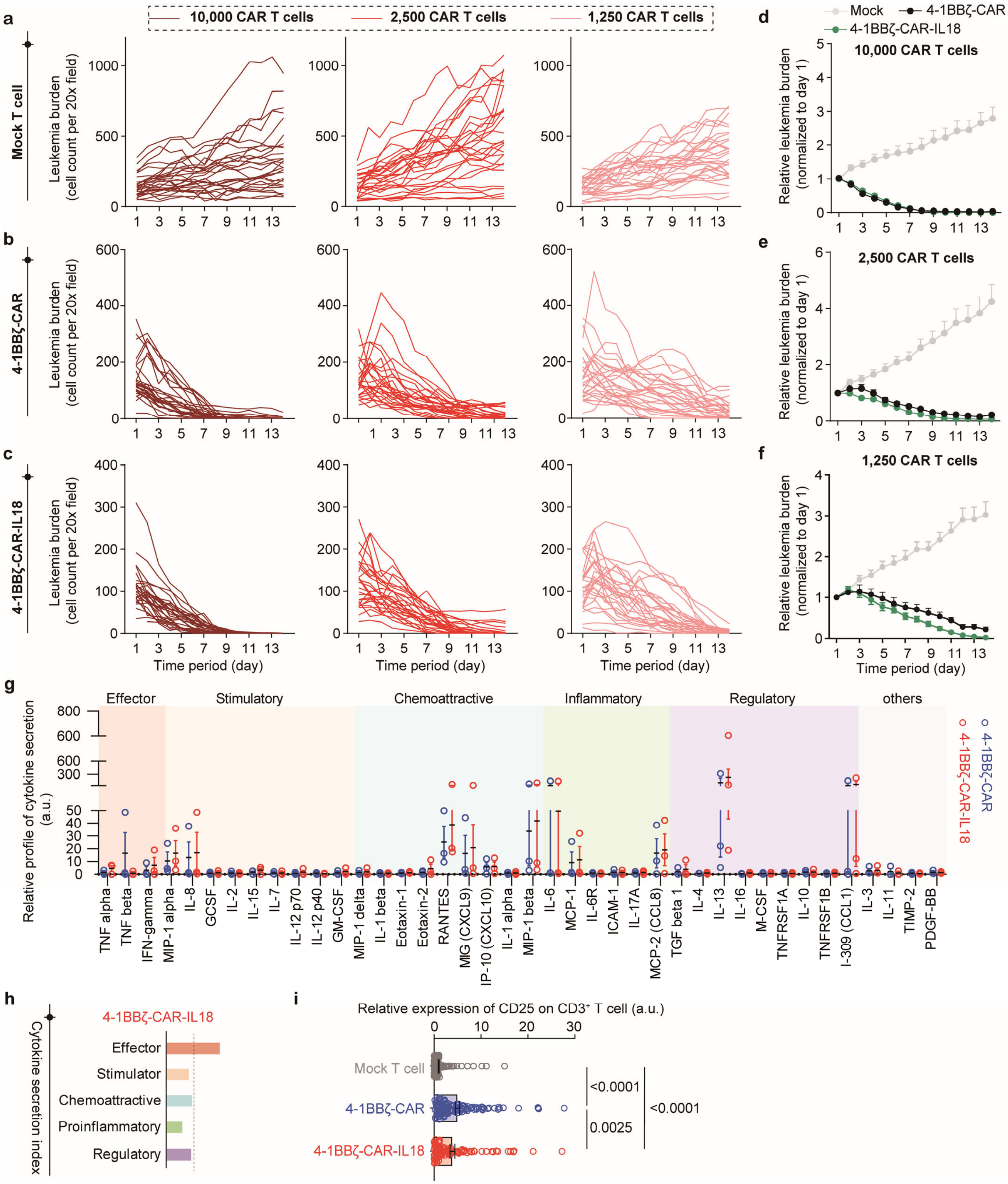

Supplementary Fig. S9. On-chip response curve under treatment of different generation CAR T cells at different doses. (**a-c**) On-chip response curves under treatments of Mock T cell (**a**), 2nd-gen 4-1BBζ-CAR (**b**), 4th-gen 4-1BBζ-CAR-IL18(**c**). Data was collected from three independent experiments (n=3) from three healthy donors, ND410, NM 11/03 and ND578 and each experiment with 2 or 3 technical replicates. (**d-f**) Comparative results of on-chip response curves under treatment of different CAR T cells

at the number of (**d**) 10,000, (**e**) 2,500 and (**f**) 1,250. Data was collected from three independent experiments (n=3), mean and s.e.m. (**g**) Profiling of relative cytokine secretion (absolute unit, a.u.) from leukemia chips treated with 4th-gen 4-1BBζ-CAR-IL18 or 2nd-gen 4-1BBζ-CAR T cells (dose of 10,000 CAR T cells) for 2 days using a Human Inflammation Array C3 membrane kit. Data was collected from three independent experiments (n=3). Data are present as mean and s.e.m. (**h**) Cytokine secretion index of 4th-gen 4-1BBζ-CAR-IL18 benchmarked by 2nd-gen 4-1BBζ-CAR (dash line). Cytokines are divided into five categories, i.e., effector, stimulatory, chemoattractive, inflammatory, and regulatory according to its role in immune response processes and present at weighted average. (**i**) Surface expression of CD25 on different CAR T cells after on-chip interaction with leukemia blasts for 2 days (absolute unit, a.u.). Data was collected from four technical replicates (n=4). One-way ANOVA followed by Dunn's multiple comparisons test, mean and s.e.m.

Supplementary Video 1. 2D time-lapse imaging showed CAR T cell extravasation, related to **Extended Data Fig. 3a**. T cells are in red, Reh B-ALL leukemia blasts are in green, and vessels (HUVECs) are in bule.

Supplementary Video 2. 2D time-lapse imaging showed CAR T cell infiltration, related to **Extended Data Fig. 3b**. T cells are in red, Reh B-ALL leukemia blasts are in green, and vessels (HUVECs) are in bule.

Supplementary Video 3. 2D time-lapse imaging showed the process of killing a Reh B-ALL blast (green) by a CAR T cell (red), related to **Extended Data Fig. 3c**.

Supplementary Video 4. Confocal time-lapse imaging showed the process of extravasation, infiltration, and killing of healthy donor-derived CAR T cell in the leukemia bone marrow chip. Vessel was formed with VE-CAD-GFP expressing HUVECs (in green). Leukemia blast was K562-meso-19-mCherry (in yellow). CAR T cells were stained with DiD dye (in red). The video was captured at 5min/frame within 14 hours using Nikon C2i confocal microscopy and a 20× objective.

Supplementary Video 5. Confocal time-lapse imaging showed the process of extravasation, infiltration, and killing of patient-derived CAR T cell in the leukemia bone marrow chip. Vessel was formed with VE-CAD-GFP expressing HUVECs (in green). Leukemia blast was K562-meso-19-mCherry (in yellow). CAR T cells were stained with DiD dye (in red). The video was captured at 5min/frame within 14 hours using Nikon C2i confocal microscopy and a 20× objective.
