# Supplementary material for: Bioengineered immunocompetent preclinical trial-on-chip tool enables screening of CAR T cell therapy for leukaemia
Source: Nat Biomed Eng. 2025 Jul 1;9(12):2098–114. doi: 10.1038/s41551-025-01428-2 (PMC12705464; doi:10.1038/s41551-025-01428-2)
Supplement: Supplementary file 2 — Reporting Summary [file 41551_2025_1428_MOESM2_ESM.pdf]

nature portfolio

Corresponding author(s): Weiqiang Chen

Last updated by author(s): Apr 6, 2025

Reporting Summary

Nature Portfolio wishes to improve the reproducibility of the work that we publish. This form provides structure for consistency and transparency in reporting. For further information on Nature Portfolio policies, see our Editorial Policies and the Editorial Policy Checklist.

Statistics

For all statistical analyses, confirm that the following items are present in the figure legend, table legend, main text, or Methods section.

n/a	Confirmed	
☐	☒	The exact sample size (*n*) for each experimental group/condition, given as a discrete number and unit of measurement
☐	☒	A statement on whether measurements were taken from distinct samples or whether the same sample was measured repeatedly
☐	☒	The statistical test(s) used AND whether they are one- or two-sided<br>*Only common tests should be described solely by name; describe more complex techniques in the Methods section.*
☒	☐	A description of all covariates tested
☐	☒	A description of any assumptions or corrections, such as tests of normality and adjustment for multiple comparisons
☐	☒	A full description of the statistical parameters including central tendency (e.g. means) or other basic estimates (e.g. regression coefficient) AND variation (e.g. standard deviation) or associated estimates of uncertainty (e.g. confidence intervals)
☐	☒	For null hypothesis testing, the test statistic (e.g. *F*, *t*, *r*) with confidence intervals, effect sizes, degrees of freedom and *P* value noted<br>*Give P values as exact values whenever suitable.*
☒	☐	For Bayesian analysis, information on the choice of priors and Markov chain Monte Carlo settings
☒	☐	For hierarchical and complex designs, identification of the appropriate level for tests and full reporting of outcomes
☒	☐	Estimates of effect sizes (e.g. Cohen's *d*, Pearson's *r*), indicating how they were calculated

Our web collection on statistics for biologists contains articles on many of the points above.

Software and code

Policy information about availability of computer code

Data collection	Zeiss Axio Observer.Z1 was used to obtain fluorescence images and time-lapse images.<br>Nikon C2i confocal microscope (Nikon NIS-Elements Microscope Imaging Software, Ar version) was used to obtain confocal images.<br>BioTek Synergy Plate Reader was used to obtain ELISA data.<br>ChemiDoc Imaging System (Bio-rad) was used to obtain chemiluminescence of membrane based assays.<br>Details are described in Methods and the relevant figure legends.
Data analysis	Graphpad Prism (version 10) was used for statistical analysis.<br>Adobe Illustrator (version 2025) was used for figure preparation.<br>BD FACSDiva software (version 5) was used for acquiring flow cytometry data and Flowjo version 10 was used for analyzing data.<br>CellRanger (version 3.1) and R package Seurat (version 4.1.1) were used for analyzing scRNA-seq data.<br>ImageJ (NIH) or Fiji (version 2) was used for image quantification.<br>Details are described in Methods and the relevant figure legends.

For manuscripts utilizing custom algorithms or software that are central to the research but not yet described in published literature, software must be made available to editors and reviewers. We strongly encourage code deposition in a community repository (e.g. GitHub). See the Nature Portfolio guidelines for submitting code & software for further information.

Data

Policy information about availability of data

All manuscripts must include a data availability statement. This statement should provide the following information, where applicable:

- Accession codes, unique identifiers, or web links for publicly available datasets
- A description of any restrictions on data availability
- For clinical datasets or third party data, please ensure that the statement adheres to our policy

Data supporting the results in this study are available within the paper and its Supplementary Information. The scRNA-seq data is available in the Gene Expression Omnibus (GEO) under accession number GSE293390. The Ensemble hg38/GRCh38 reference genome for scRNA-seq read alignment is available on NCBI Datasets (GCF_000001405.40). The raw and analyzed datasets generated during the study are available from the corresponding author on reasonable request. Source data are provided with this paper.

Research involving human participants, their data, or biological material

Policy information about studies with human participants or human data. See also policy information about sex, gender (identity/presentation), and sexual orientation and race, ethnicity and racism.

Reporting on sex and gender	N/A
Reporting on race, ethnicity, or other socially relevant groupings	N/A
Population characteristics	N/A
Recruitment	The leukemia patient 4-1BBζ-CAR19-IL18 T cells, a third-generation CAR T cells, were obtained from the CART19-IL18 clinical trial at the University of Pennsylvania (ClinicalTrials.gov: NCT04684563). In this context, autologous T cells were transduced with a humanized anti-CD19 CAR lentiviral vector, that was engineered to co-express IL-18. The study was approved by the Institutional Review Board at the University of Pennsylvania. It was conducted in accordance with the principles of the Declaration of Helsinki. All patients provided written informed consent. To ensure compliance with the HIPAA regulations, all of the samples were deidentified before analysis on-chip.
Ethics oversight	The current study involved a secondary investigation of patient 4-1BBζ-CAR19-IL18 T-cells collected from an existing clinical trial for which the University of Pennsylvania Institutional Review Board provided insight.

Note that full information on the approval of the study protocol must also be provided in the manuscript.

Field-specific reporting

Please select the one below that is the best fit for your research. If you are not sure, read the appropriate sections before making your selection.

☒ Life sciences ☐ Behavioural & social sciences ☐ Ecological, evolutionary & environmental sciences

For a reference copy of the document with all sections, see nature.com/documents/nr-reporting-summary-flat.pdf

Life sciences study design

All studies must disclose on these points even when the disclosure is negative.

Sample size	No predetermination of sample size was conducted by statistical method. A minimum of 2 biological replicates or 3 technical replicates were used for all experiments, unless stated otherwise (sample sizes for each experiment are stated in the corresponding figure captions). All the experiments were performed in n greater than or equal to 3 so as to provide sufficient statistical power to discern statistically significant differences as indicated in the each figure.
Data exclusions	No data were excluded from the experiments.
Replication	All experiments were reliably replicated. Experiments were performed on different days, using different batches of chips, and with different healthy donors and patients samples (the exact sample sizes of all experiments are stated at the corresponding figure legends), indicating high robustness. All experiments were analysed from at least 3 technical replicates, unless stated otherwise.
Randomization	No randomization was used because the study was focused on validating the chip performance rather than evaluating and comparing clinical samples.
Blinding	The investigators were not blinded during data collection and analysis.

Reporting for specific materials, systems and methods

We require information from authors about some types of materials, experimental systems and methods used in many studies. Here, indicate whether each material, system or method listed is relevant to your study. If you are not sure if a list item applies to your research, read the appropriate section before selecting a response.

Materials & experimental systems

n/a	Involved in the study
☐	☒ Antibodies
☐	☒ Eukaryotic cell lines
☒	☐ Palaeontology and archaeology
☒	☐ Animals and other organisms
☒	☐ Clinical data
☒	☐ Dual use research of concern
☒	☐ Plants

Methods

n/a	Involved in the study
☒	☐ ChIP-seq
☐	☒ Flow cytometry
☒	☐ MRI-based neuroimaging

Antibodies

Antibodies used

Primary antibodies for characterizing specific cell types:

vascular cell (FITC anti-human CD31, Clone WM59, Cat#303104, BioLegend; PE anti-human CD31, Clone WM59, Cat#303106, BioLegend; Alexa Fluor 488 anti-human CD54 (ICAM-1), Clone HA58, Cat#353129, BioLegend; PE anti-human VE-cadherin, Clone BV9, Cat#348506, BioLegend), all at 1:50 dilution.

bone marrow mesenchymal stem cell (APC anti-human CD90/Thy1,Clone 5E10, Cat#328113, BioLegend), 1:50 dilution.

hematopoietic cell (APC anti-human CD45,Clone HI30, Cat#304012, BioLegend), 1:50 dilution.

hematopoietic stem cells (APC anti-human CD34, Clone 581, Cat#343510, BioLegend), 1:50 dilution.

T cell (CD3, FITC anti-human CD3, Clone OKT3, Cat#317306, BioLegend; PE anti-human CD3, Clone OKT3, Cat#317308, BioLegend; APC anti-human CD3, Clone UCHT1, Cat#300412, BioLegend; CD4, PE anti-human CD4, Clone OKT4, Cat#317410, BioLegend; CD8, FITC anti-human CD8a, Clone HIT8a, Cat#300906, BioLegend; PE anti-human CD8a, Clone HIT8a, Cat#300908, BioLegend; APC anti-human CD8a, Clone HIT8a, Cat#300912, BioLegend), all at 1:20 dilution.

monocyte (Alexa Fluor 647 anti-human CD14, Clone HCD14, Cat#325612, BioLegend; PE anti-human HLA-DR, Clone L243, Cat#307606, BioLegend), macrophage (Alexa Fluor 488 anti-human CD68, Clone Y1/82A, Cat#333812, BioLegend), all at 1:50 dilution.

Antibodies for characterizing T cell proliferation: APC anti-human Ki67, Clone Ki-67, Cat#350514, Alexa Fluor 488 anti-human Ki67,Clone Ki-67, Cat#350508, BioLegend and T cell activation: PE anti-human CD69 (Clone FN50, Cat#310906, BioLegend), PE anti-human CD25 (Clone BC96, Cat#302606, BioLegend), or PE anti-human granzyme B (Clone QA16A02, Cat#372208, BioLegend), all at 1:50 dilution.

To indicate CAR T cell, biotinylated monoclonal anti-FMC63 scFv (CAR) antibody (Clone Y45, Cat#FM3-BY54, ACRO Biosystems) was used, followed by incubation with PE (Cat#405245, BioLegend) or APC (Cat#405243, BioLegend) conjugated Streptavidin, or directly stained with FITC-Labeled Monoclonal Anti-FMC63 scFv Antibody (1:50, Clone Y45, Cat#FM3-FY45P1, ACRO Biosystems) or APC-Labeled Monoclonal Anti-FMC63 scFv Antibody (1:50, Clone Y45, Cat#FM3-AY54P1, ACRO Biosystems). 4',6-diamidino-2-phenylindole (DAPI, Cat#D1306, Thermo Fisher Scientific) was used to counterstain nuclei.

Antibodies for characterizing extracellular matrices: DyLight488-Laminin (Polyclonal, Cat#PA522901, Thermo Fisher Scientific), PE-Fibronectin (Clone P1H11, Cat# IC1918P, R&D Systems), and Alexa Fluor 647-Collagen IV (Clone 1042, Cat#51-9871-80, Thermo Fisher Scientific), all at 1:50 dilution.

Sample hashing (1:250)
Hashtag1-GTCAACTCTTTAGCG, BioLegend, Clone LNH-94; 2M2, Cat#394601; Hashtag2-TGATGGCCTATTGGG, BioLegend, Clone LNH-94; 2M2, Cat#394603, Hashtag3-TTCCGCCTCTCTTTG, BioLegend, Clone LNH-94; 2M2, Cat#394605, Hashtag4-AGTAAGTTCAGCGTA, BioLegend, Clone LNH-94; 2M2, Cat#394607.

Validation

All antibodies were validated by the manufacturers and used according to the manufacturers' protocols.

https://www.biolegend.com/en-us/products/fitc-anti-human-cd31-antibody-881
https://www.biolegend.com/en-us/products/pe-anti-human-cd31-antibody-882
https://www.biolegend.com/en-us/products/alexa-fluor-488-anti-human-cd54-antibody-18741
https://www.biolegend.com/en-us/products/pe-anti-human-cd144-ve-cadherin-antibody-6615

https://www.biolegend.com/en-us/products/apc-anti-human-cd90-thy1-antibody-4116

https://www.biolegend.com/en-us/products/apc-anti-human-cd45-antibody-705

https://www.biolegend.com/en-us/products/apc-anti-human-cd34-antibody-6090

https://www.biolegend.com/en-us/products/fitc-anti-human-cd3-antibody-3644
https://www.biolegend.com/en-us/products/pe-anti-human-cd3-antibody-3645
https://www.biolegend.com/en-us/products/apc-anti-human-cd3-antibody-861
https://www.biolegend.com/en-us/products/pe-anti-human-cd4-antibody-3654
https://www.biolegend.com/en-us/products/fitc-anti-human-cd8a-antibody-761
https://www.biolegend.com/en-us/products/pe-anti-human-cd8a-antibody-762
https://www.biolegend.com/en-us/products/apc-anti-human-cd8a-antibody-759

https://www.biolegend.com/en-us/products/alexa-fluor-647-anti-human-cd14-antibody-3955
https://www.biolegend.com/en-us/products/pe-anti-human-hla-dr-antibody-790
https://www.biolegend.com/en-us/products/alexa-fluor-488-anti-human-cd68-antibody-6543

https://www.biolegend.com/en-us/products/alexa-fluor-488-anti-human-ki-67-antibody-7275
https://www.biolegend.com/en-us/products/apc-anti-human-ki-67-antibody-7531

hhttps://www.biolegend.com/en-us/products/pe-anti-human-cd69-antibody-1672
https://www.biolegend.com/en-us/products/pe-anti-human-cd25-antibody-616
https://www.biolegend.com/en-us/products/pe-anti-human-mouse-granzyme-b-recombinant-antibody-14431

https://www.acrobiosystems.com/P3591-Biotinylated-Monoclonal-Anti-FMC63-Antibody-Mouse-IgG1-Avitag%E2%84%A2-%28Y45%29.html?srsltid=AfmBOorZIo7U8-saSRvFAU8N1xCvmAhUilqshDzhAAVCgk8tTXe3eyXF
https://www.acrobiosystems.com/P4572-FITC-Labeled-Monoclonal-Anti-FMC63-Antibody-Mouse-IgG1-%28Y45%29-%28HEK293%29.html
https://www.acrobiosystems.com/P5006-APC-Labeled-Monoclonal-Anti-FMC63-Antibody-Mouse-IgG1-%28Y45%29-%28Site-specific-conjugation%29.html?srsltid=AfmBOorTWFk3oF5VnynQ4GUA0PcSOD2pVBtHtK62flUZwgg4fqGRQqB2

https://www.thermofisher.com/antibody/product/Laminin-Antibody-Polyclonal/PA5-22901
https://www.rndsystems.com/products/human-fibronectin-pe-conjugated-antibody-p1h11_ic1918p
https://www.thermofisher.com/antibody/product/Collagen-IV-Antibody-clone-1042-Monoclonal/51-9871-82

https://www.biolegend.com/en-us/products/totalseq-a0251-anti-human-hashtag-1-16080
https://www.biolegend.com/en-us/products/totalseq-a0252-anti-human-hashtag-2-antibody-16081
https://www.biolegend.com/en-us/products/totalseq-a0253-anti-human-hashtag-3-antibody-16084
https://www.biolegend.com/en-us/products/totalseq-a0254-anti-human-hashtag-4-antibody-16086

https://www.biolegend.com/en-us/products/pe-streptavidin-high-concentration-10067
https://www.biolegend.com/en-us/products/apc-streptavidin-high-concentration-10081

Eukaryotic cell lines

Policy information about cell lines and Sex and Gender in Research

Cell line source(s)

Leukemia cells: Human B-ALL cell line (Reh, Cat#CRL-8286, ATCC), GFP-expressing Reh cell line (Cat#T3959, Applied Biological Materials); K562-meso-19, K562-meso-19-GFP and K562-meso-19-GFP B-ALL cell lines were provided by Dr. Saba Ghassemi's lab; GFP-expressing and CD19-KO mCherry expressing Reh B-ALL cell lines were generated following the protocol described in our previous study.

Niche cells: Primary human umbilical endothelial cell (HUVEC, Cat#C2519A, Lonza), RFP-expressing HUVEC (Cat#cAP-0001RFP, Angio-Proteomie), human mesenchymal stem cell (Cat#PT-2501, Lonza), normal human lung fibroblast (Cat#CC-2512, Lonza), human fetal osteoblastic cell line (hFOB1.19, Cat#CRL-11372, ATCC), bone marrow mononuclear cells (Cat#70001.1, STEMCELL Technologies).

CAR T cells: Human anti-CD19 scFv-4-1BB-CD3ζ CAR (4-1BBζ-CAR, for short) T cells from healthy donors and cancer patients (PD145, PD323, PD356, and PD674) or non-engineered (Mock) T cells are purchased from ProMab Biotechnologies by a customized order with expansion for about 10 days. 2nd-gen human anti-CD19 CAR T cells [i.e., CD28ζ-CAR, ICOSζ-CAR, 4-1BBζ-CAR, and D3 and D9 4-1BBζ-CAR (D3-CAR and D9-CAR)], 4th-gen 4-1BBζ-CAR-IL18 T cells, and leukemia patients 4-1BBζ-CAR T cells (PD7607, PD7814, and PD8009), as well as respective Mock T cells, were prepared by Dr. Saba Ghassemi's lab at University of Pennsylvania School of Medicine with expansion for about 10 days. Leukemia patients anti-CD19 4-1BBζ-CAR T cells and autologous 4-1BBζ-CAR-IL18 T cells were obtained from a huCART19-IL18 clinical trial at the University of Pennsylvania (ClinicalTrials.gov: NCT04684563). 4-1BBζ-CAR-IL18 T cells were produced with a humanized anti-CD19 CAR lentiviral vector that was engineered to co-express IL-18. The study was approved by the Institutional Review Board at the University of Pennsylvania. It was conducted in accordance with the principles of the Declaration of Helsinki. All patients provided written informed consent. To ensure compliance with the HIPAA regulations, all of the samples were deidentified before analysis on-chip.

Authentication

Cell lines were authenticated by the providers with cell-specific markers, morphology, STR profiling, or Karyotyping.

Mycoplasma contamination

All cells were tested negative for mycoplasma contamination according to the providers and were routinely monitored for mycoplasma contamination.

Commonly misidentified lines
(See ICLAC register)

The cell lines used in this study are not on the list of commonly misidentified cell lines in ICLAC.

Flow Cytometry

Plots

Confirm that:

☒ The axis labels state the marker and fluorochrome used (e.g. CD4-FITC).

☒ The axis scales are clearly visible. Include numbers along axes only for bottom left plot of group (a 'group' is an analysis of identical markers).

☒ All plots are contour plots with outliers or pseudocolor plots.

☒ A numerical value for number of cells or percentage (with statistics) is provided.

Methodology

Sample preparation	Samples were first prepared by off-chip recovery with nattokinase (50 Fu/mL, NSK-SD, Pure Encapsulations) and then respectively incubated with antibodies for 30 minutes at 4°C. Cells were washed three times and stained with LIVE/DEAD Fixable Aqua Dead Cell Stain Kit (1:1500, Cat#L34957, Thermo Fischer Scientific) to mark live/dead cells.
Instrument	BD Biosciences LSRII UV Flow Cytometer
Software	Data was collected using BD FACSDiva Software (version 5) and analysis was performed using Flowjo software (Tree Star Inc. version 10).
Cell population abundance	Cell counts of >100,000 events were collected.
Gating strategy	Fluorescence compensations were prepared by incubating respective antibodies with CompBead Anti-Mouse Ig, κ/Negative Control Particles Set (Cat#552843, BD Biosciences). All T cells were gated as live CD3+ population.

☒ Tick this box to confirm that a figure exemplifying the gating strategy is provided in the Supplementary Information.
